# Targeting specific kinase substrates rescues increased colitis severity induced by the Crohn’s disease–linked LRRK2-N2081D variant

**DOI:** 10.1172/JCI190017

**Published:** 2025-10-01

**Authors:** George R. Heaton, Xingjian Li, Xianting Li, Xiaoting Zhou, Yuanxi Zhang, Duc Tung Vu, Marc Oeller, Ozge Karayel, Quyen Q. Hoang, Meltem Ece Kars, Nitika Kamath, Minghui Wang, Leonid Tarassishin, Matthias Mann, Inga Peter, Zhenyu Yue

**Affiliations:** 1 Center for Parkinson’s Disease Neurobiology, Departments of Neurology and Neuroscience, The Friedman Brain Institute, Icahn School of Medicine at Mount Sinai, New York, New York, USA.; 2 Proteomics and Signal Transduction, Max Planck Institute of Biochemistry, Martinsried, Germany.; 3 Department of Biochemistry and Molecular Biology, Indiana University School of Medicine, Indianapolis, Indiana, USA.; 4 The Charles Bronfman Institute for Personalized Medicine, and; 5 Department of Genetics and Genomic Sciences, Icahn School of Medicine at Mount Sinai, New York, New York, USA.

**Keywords:** Gastroenterology, Genetics, Inflammatory bowel disease, Parkinson disease

## Abstract

LRRK2 contains a kinase domain where the N2081D Crohn’s disease (CD) risk and the G2019S Parkinson’s disease (PD) pathogenic variants are located. It is not clear how the N2081D variant increases CD risk or how these adjacent mutations give rise to distinct disorders. To investigate the pathophysiology of the CD-linked LRRK2 N2081D variant, we generated a knock-in (KI) mouse model and compared its effects with those of the LRRK2-G2019S mutation. *Lrrk2^N2081D^* KI mice demonstrated heightened sensitivity to induced colitis, resulting in more severe intestinal damage than in *Lrrk2^G2019S^* KI and WT mice. Analysis of colon tissue revealed distinct mutation-dependent LRRK2 RAB substrate phosphorylation, with significantly elevated phosphorylated RAB10 levels in *Lrrk2^N2081D^* mice. In cells, we demonstrated that the N2081D mutation activates LRRK2 through a mechanism distinct from that of LRRK2-G2019S. We also found that proinflammatory stimulation enhances LRRK2 kinase activity, leading to mutation-dependent differences in RAB phosphorylation and inflammatory responses in dendritic cells (DCs). Finally, we show that knockout of *Rab12*, but not pharmacological LRRK2 kinase inhibition, significantly reduced colitis severity in *Lrrk2^N2081D^* mice. Our study characterizes the pathogenic mechanisms of LRRK2-linked CD, highlights structural and functional differences between disease-associated LRRK2 variants, and suggests RAB proteins as promising therapeutic targets for modulating LRRK2 activity in CD treatment.

## Abstract 2

Crohn’s disease-linked LRRK2 mutations target specific kinase substrates and worsen colitis in a newly developed mouse model.

## Introduction

Mutations in *LRRK2* are a major cause of Parkinson’s disease (PD), a common age-related neurodegenerative disorder characterized by the degeneration of dopaminergic neurons in the substantia nigra pars compacta and the presence of aggregated α-synuclein in neurons (1, 2). The *LRRK2* gene encodes leucine-rich repeat kinase 2 (LRRK2), a large, complex protein with multiple functional regions (3). The catalytic core of LRRK2 consists of a GTPase and an adjacent serine/threonine kinase domain. LRRK2 can autophosphorylate at serine residue S1292 in its leucine-rich repeat (LRR) region and phosphorylate RAB GTPases at a conserved site within their switch II domain (4). A growing body of evidence suggests that LRRK2 plays a significant role in regulating membrane trafficking at various stages of the endolysosomal pathway, including a response to damaged lysosomes through its interaction with and phosphorylation of RAB GTPases (4–10).

Our previous exome-wide association analysis of Crohn’s disease (CD) in individuals of Ashkenazi Jewish descent identified the N2081D coding variant in LRRK2 as a CD risk allele (3). CD is a type of inflammatory bowel disease (IBD), a chronic disorder affecting the gastrointestinal tract (11). In our present study, we confirmed the genetic pleiotropy of LRRK2 in both CD and PD through association analysis of 3 large biobank data sets. To investigate the mechanistic basis of these observations—specifically how the N2081D variant affects LRRK2 function to increase CD risk and how its differences with the PD-associated G2019S variant might explain their distinct phenotypes—we generated a knock-in (KI) mouse model carrying the LRRK2 N2081D mutation using CRISPR/Cas9 gene editing.

We found that *Lrrk2^N2081D^* KI mice have significantly increased intestinal inflammation and colitis severity, confirming the N2081D variant as a pathogenic risk factor. Comparative tissue analysis from *Lrrk2^G2019S^* and *Lrrk2^N2081D^* mutation carriers revealed significant differences in LRRK2 RAB substrate phosphorylation, demonstrating distinct functional effects of these variants. Mechanistically, we found that the N2081D variant activates LRRK2 by disrupting an interdomain interaction between the LRR and kinase regions. Furthermore, proinflammatory stimulation enhances LRRK2 kinase activity, leading to mutation-dependent differences in RAB phosphorylation and inflammatory responses.

Finally, we demonstrated that genetic targeting of specific LRRK2 substrates reduces colitis severity in *Lrrk2^N2081D^* mutation carriers, whereas a small molecule LRRK2 inhibitor did not produce a clear therapeutic effect. Our findings highlight distinct pathological features of the LRRK2-N2081D variant and suggest potential therapeutic targets for CD treatment.

## Results

### Association analysis of CD- and PD-linked variants confirms genetic pleiotropy of LRRK2.

We examined the associations of the LRRK2 G2019S and N2081D variants with CD and PD across 3 biobanks: Bio*Me* BioBank, UK Biobank (Genebass), and the VA Million Veteran Program (MVP) (12, 13). We conducted population-specific association tests and 4 meta-analyses, including a multi-ancestry meta-analysis that comprised up to 1,072,253 individuals.

**Figure 1:**
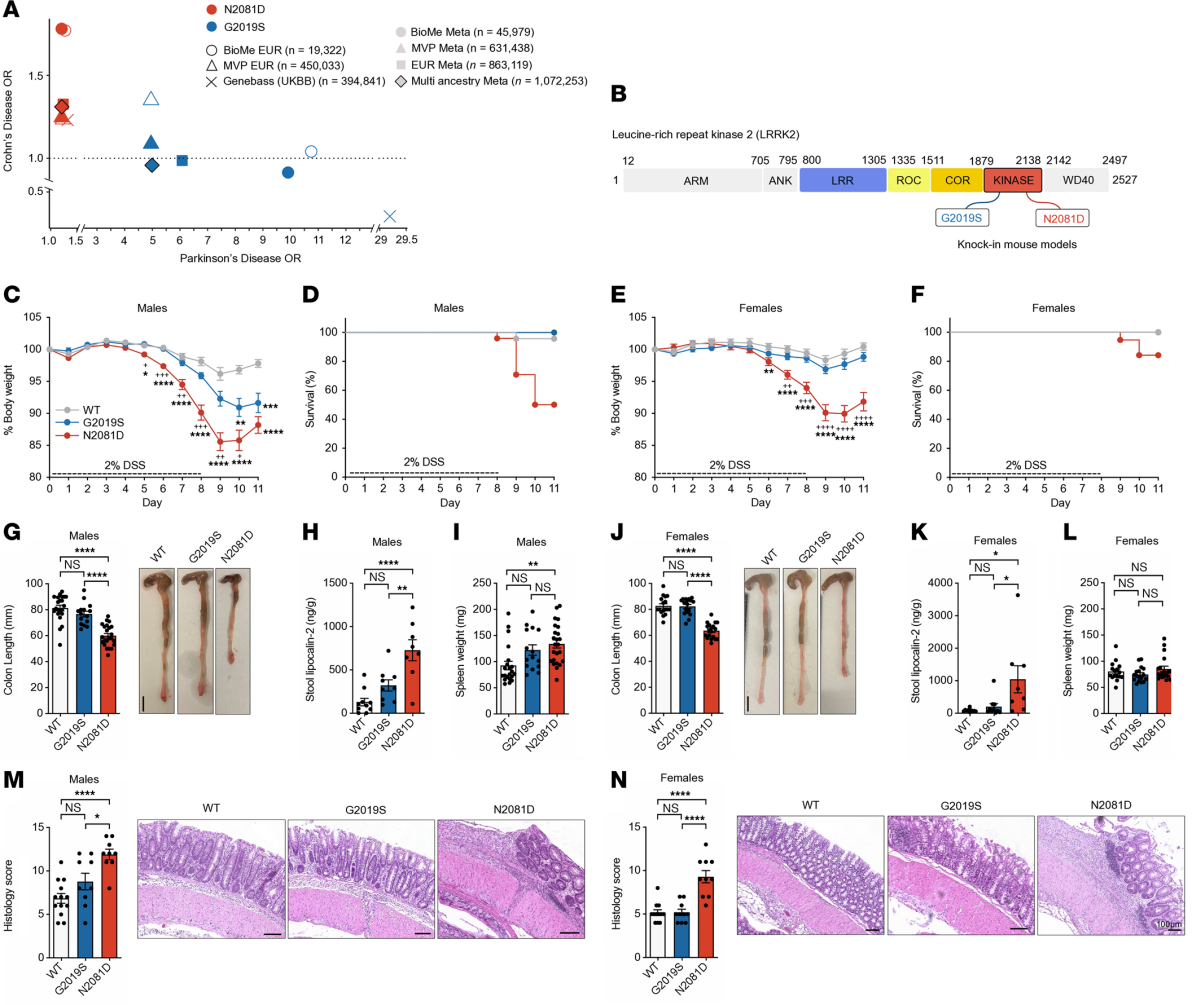
Validation of the LRRK2-N2081D KI mouse model of CD. (**A**) Odds ratios (ORs) for the associations of the G2019S and N2081D LRRK2 variants with CD and PD. (**B**) Schematic representation of the LRRK2 protein, highlighting the protein domains and locations of disease-associated variants. (**C** and **E**) Normalized percentage of body weight during acute DSS treatment in male and female mice (male mice: *n =* 22 WT, *n =* 15 G2019S, *n =* 24 N2081D; female mice: *n =* 17 each WT and G2019S, *n =* 19 N2081D). * indicates a significant difference between WT and either G2019S or N2081D; + indicates a significant difference between N2081D and G2019S. (**D** and **F**) Survival curves of male and female mice. Animals were sacrificed when humane endpoints were surpassed. Male mice showed significantly different survival among genotypes (****P < 0.0001); female mice showed no significant differences. (**G** and **J**) Measurements of colon length and representative images of male and female mice, respectively. (**H** and **K**) Stool lipocalin-2 measured by ELISA in male and female mice (male mice: *n =* 10 WT, *n =* 9 G2019S, *n =* 8 N2081D; female mice: *n =* 10 each WT and G2019S, *n =* 8 N2081D). (**I** and **L**) Spleen weight recorded at sacrifice for male and female mice (male mice: *n =* 21 WT, *n =* 15 G2019S, *n =* 24 N2081D; female mice: *n =* 16 WT, *n =* 17 G2019S, *n =* 19 N2081D). (**M** and **N**) Representative images and histological scoring of intestinal inflammation and damage in male and female mice (male mice: *n =* 13 WT, *n =* 9 each G2019S and N2081D; female mice: *n =* 12 WT, *n =* 10 each G2019S and N2081D). One-way ANOVA with Tukey’s post hoc test was used for **C**, **E**, **F**, and **H**–**N**; log-rank (Mantel-Cox) test was used for survival curves in **D** and **F**. *P < 0.05, **P < 0.01, ***P < 0.001, ****P < 0.0001. EUR, European; meta, meta-analysis; UKBB, UK Biobank.

Our analysis uncovered significant differences in the ORs of these LRRK2 variants for CD and PD (Figure 1A and Supplemental Tables 1 and 2; supplemental material available online with this article; https://doi.org/10.1172/JCI190017DS1). Specifically, the N2081D variant increases the risk for both CD and PD, whereas the G2019S variant was significantly associated with an increased risk for PD in all European-specific analyses and meta-analyses. Furthermore, G2019S increased the risk of CD in the European-only analyses of Bio*Me* BioBank and MVP and the meta-analysis of MVP. However, its association with CD in the UK Biobank was inconsistent with previous reports; none of the CD cases in the UK Biobank carried the G2019S variant, likely due to significant differences in G2019S risk allele frequencies in various ancestral groups. Overall, these findings further demonstrate the extent of the genetic pleiotropy between CD and PD within the LRRK2 gene (3, 14).

### LRRK2^N2081D^ mice have increased sensitivity to chemically induced colitis.

We developed a KI mouse model of the LRRK2-N2081D variant, using CRISPR/Cas9 (Figure 1B and Supplemental Figure 1A). This model carries the *Lrrk2^N2081D^* murine equivalent of the human N2081D LRRK2 variant, expressed at endogenous levels. Additionally, we used a previously established *Lrrk2* KI model with the G2019S mutation (*Lrrk2^G2019S^*) (15). Homozygous N2081D KI mice appeared grossly normal compared with WT and *Lrrk2^G2019S^* mice (Supplemental Figure 1, B–D).

To evaluate the impact of LRRK2 mutations on colonic inflammation and colitis symptoms, we used the dextran sodium sulfate (DSS**)** model. Prolonged DSS exposure leads to epithelial cell death and compromised intestinal barrier integrity, resulting in leukocyte infiltration and colonic tissue damage (16). We induced colitis by administering 2% DSS in the drinking water to 16-week-old male and female WT mice and homozygous carriers of the *Lrrk2^G2019S^* or *Lrrk2^N2081D^* variant for 8 days, followed by 3 days of facility water for partial recovery.

In both sexes, *Lrrk2^N2081D^* mice exhibited more severe weight loss compared with WT and *Lrrk2^G2019S^* mice (Figure 1, C and E). Male mice experienced the most severe weight loss, with a significant number of N2081D male mice requiring early sacrifice due to colitis severity (Figure 1D). At our experimental end point, both male and female *Lrrk2^N2081D^* mice had shorter colons than their sex-matched WT and *Lrrk2^G2019S^* counterparts, indicating a greater degree of colonic inflammation (Figure 1, G and J). Consistent with these results, stool samples extracted from colons of N2081D mutation carriers had significantly higher levels of the proinflammatory marker lipocalin-2 (Figure 1, H and K). Male N2081D carriers also had increased spleen weight compared with WT mice, suggesting a heightened immune response (Figure 1, I and L).

Histological examination of colon samples revealed a greater degree of inflammation and tissue damage in both male and female *Lrrk2^N2081D^* carriers compared with sex-matched *Lrrk2^G2019S^* and WT mice (Figure 1, M and N). Collectively, these results show that the *Lrrk2^N2081D^* mutation significantly exacerbates colonic inflammation and the severity of induced colitis, validating the *Lrrk2^N2081D^* model as a tool to study LRRK2-linked CD pathogenic mechanisms.

### Distinct functional effects of CD- and PD-linked LRRK2 variants.

**Figure 2:**
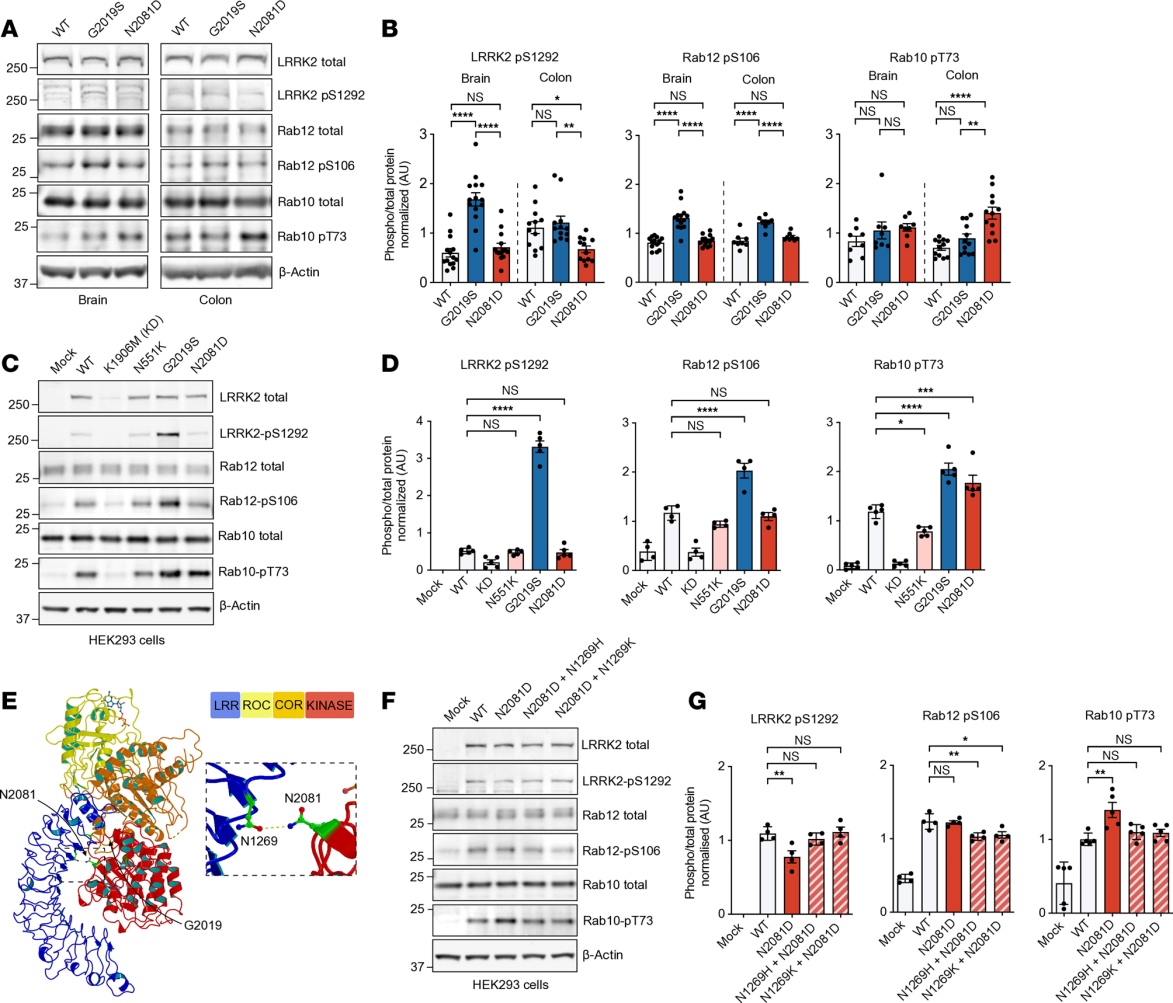
Kinase activity characterization of PD- and CD-linked LRRK2 variants. (**A**) Representative IBs of colon and brain tissue lysates from WT, G2019S, and N2081D KI mice. (**B**) Quantification of endogenous LRRK2 S1292 autophosphorylation and Rab substrate phosphorylation. (**C**) Representative IBs of HEK293 cells expressing LRRK2 variants. (**D**) Quantification of LRRK2 S1292 autophosphorylation and phosphorylation of endogenous Rab substrates in HEK293 cells (*n* = 5 independent experiments). (**E**) Structural modeling of LRRK2 domains (LRR, ROC, COR, and kinase) based on the full-length structure, with the positions of N2081 and G2019 residues highlighted. (**F**) Representative IBs of HEK293 cells expressing LRRK2 mutants at N2081 and N1269 residues. (**G**) Quantification of LRRK2 autophosphorylation and Rab phosphorylation (*n* = 5 independent experiments). One-way ANOVA with Tukey’s post hoc test was used for **B**; 1-way ANOVA with Dunnett’s post hoc test was used for **D** and **G**. *P < 0.05, **P < 0.01, ***P < 0.001, ****P < 0.0001.

To assess the effects of the G2019S and N2081D mutations on LRRK2 kinase activity, we first measured levels of established LRRK2-kinase targets in brain and colon tissues of KI mouse models. Analysis of male mice approximately 3 months old revealed elevated levels of the phosphorylated LRRK2-S1292 in *Lrrk2^G2019S^* mutation carriers, with significant differences observed in the brain compared with WT and *Lrrk2^N2081D^* animals. Notably, increased phosphorylation of RAB12 was exclusively observed in *Lrrk2^G2019S^* mice in both brain and colon tissue. However, LRRK2-G2019S only modestly increased RAB10 phosphorylation levels compared with WT (Figure 2, A and B).

In contrast, N2081D mutation carriers had significantly higher levels of phosphorylated RAB10 in colon tissues, with a similar trend in the brain, compared with WT and G2019S mice (Figure 2, A and B). A comparison of brain and colon samples from N2081D mutation carriers showed significantly higher levels of phosphorylated RAB10 in the colon than in the brain, which may suggest that this variant has a stronger effect in this tissue (Supplemental Figure 2, A and B). Analysis of a subset of N2081D male mice with severe colitis symptoms showed a striking increase in phosphorylated RAB10 in DSS-treated colon tissue, compared with untreated animals, indicating the N2081D mutation’s effects may be further amplified by severe inflammation (Supplemental Figure 2, C and D). Overall, our findings suggest that although both the N2081D and G2019S mutations enhance LRRK2 kinase activity, their distinct patterns of substrate phosphorylation indicate different mechanisms of action.

To further investigate the functional effects of CD- and PD-linked LRRK2 variants, we assessed their impact on LRRK2 kinase activity in HEK293 cells (Figure 2, C and D). Mock transfection and the kinase-dead K1906M variant served as negative controls. Consistent with our observations in mouse tissue, only LRRK2-G2019S significantly enhanced LRRK2-S1292 autophosphorylation. Increased RAB12-S106 phosphorylation was also observed only following LRRK2-G2019S expression. Both the N2081D and G2019S variants induced significant increases in RAB10 phosphorylation, whereas the CD-protective N551K variant decreased RAB10 phosphorylation levels (Figure 2, C and D). We further validated the effect of the N2081D mutation using confocal microscopy, quantifying increased phosphorylated RAB10 levels relative to WT and mock-transfected cells (Supplemental Figure 3, A and B).

To investigate the mechanism of the N2081D mutation, we examined previously reported high-resolution structural models of inactive, full-length LRRK2 (Figure 2E) (17). These models predict a hydrophilic interaction between the kinase domain’s N2081 residue and the N1269 residue in the LRR domain. In its inactive state, the LRR domain tightly interacts with the kinase domain, blocking the active site. The N2081D mutation is expected to disrupt this interaction, exposing the kinase domain and allowing greater access to RAB substrates. To validate this proposed mechanism, we introduced mutations at the N1269 residue to re-establish its interaction with the N2081D mutant and evaluated the impact on LRRK2’s kinase activity. Our data show that the N1269H and N1269K mutations effectively counteracted N2081D-mediated activation of LRRK2, reducing RAB10 phosphorylation to near WT levels (Figure 2, F and G). Although LRRK2-N2081D alone did not increase RAB12 phosphorylation, the compensatory N1269 mutations also decreased RAB12 phosphorylation relative to the WT. Additionally, the N2081D variant caused a modest decrease in S1292 phosphorylation compared with the WT. This reduction likely results from the disruption of the N1269-N2081 interaction, causing separation between the S1292 residue in the LRR domain and the kinase domain’s active site. Supporting this, the N1269 mutations restored S1292 phosphorylation to WT levels in the presence of the N2081D variant (Figure 2, F and G).

In summary, our findings suggest the N2081D mutation activates LRRK2 by inducing a conformational shift, resulting in functional effects distinct from the PD-associated LRRK2 G2019S variant.

### DC stimulation induces LRRK2 mutation-dependent differences in RAB phosphorylation and inflammatory responses.

**Figure 3:**
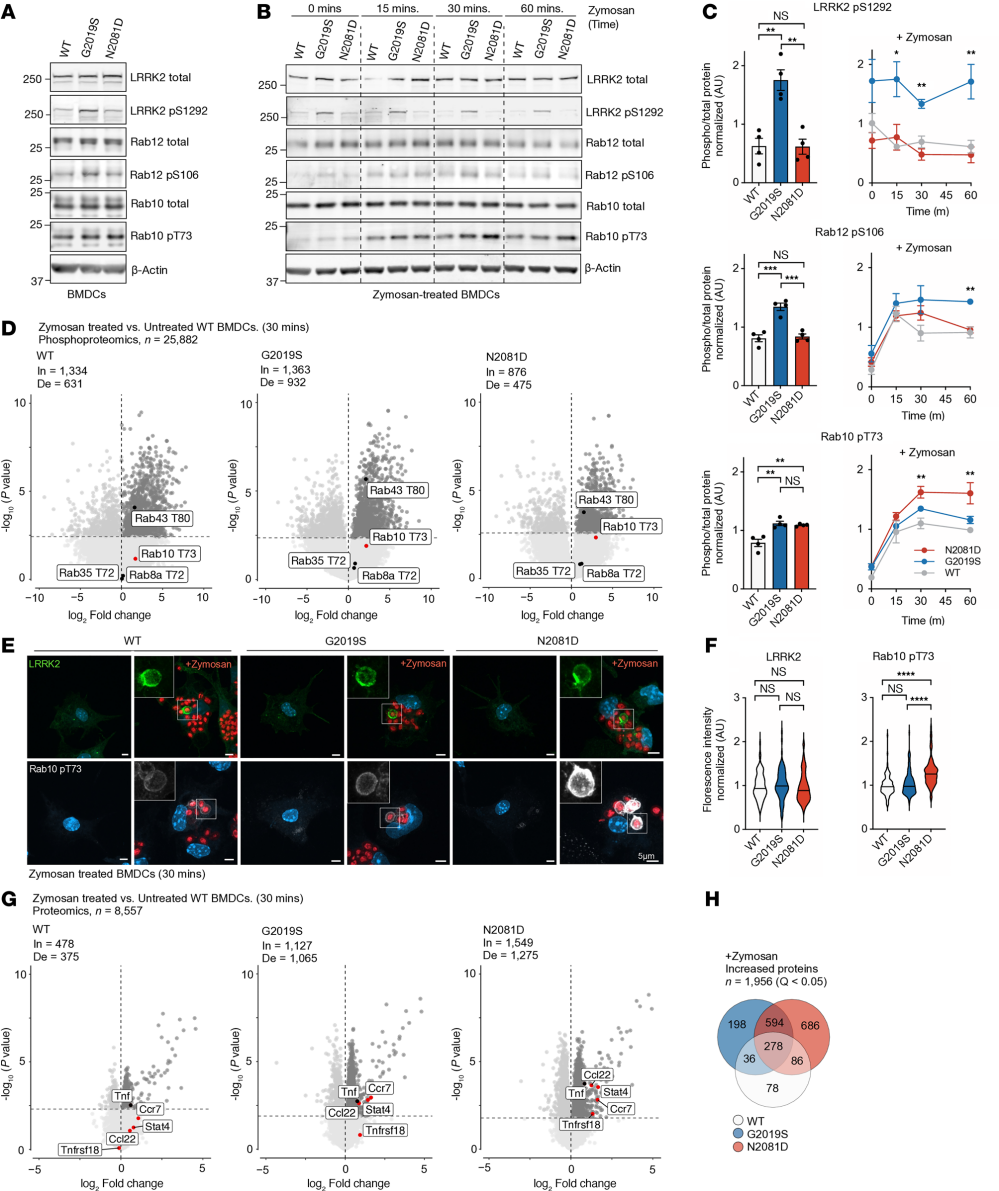
Activation of DCs induces LRRK2 mutation–dependent differences in RAB phosphorylation and inflammatory signaling. (**A**) Representative IB of untreated WT, G2019S, and N2081D BMDCs (*n* = 4 independent experiments). (**B**) Representative IB of WT, G2019S, and N2081D BMDCs treated with zymosan for 60 minutes (*n* = 4 independent experiments). (**C**) Quantification of IBs from untreated and zymosan-treated BMDCs. (**D**) Phosphoproteomics volcano plot of WT, G2019S, and N2081D BMDCs treated with zymosan for 30 minutes; established LRRK2-phosphorylated RABs are annotated. The horizontal dashed line indicates a *q*-value threshold of < 0.05. BMDC cultures were derived from 8 animals per genotype and 8 independent experiments. De, number of proteins with significantly decreased phosphorylation; In, number of proteins with significantly increased phosphorylation. (**E**) Confocal images of endogenous LRRK2 and phosphorylated RAB10 after 30 minutes of zymosan treatment. (**F**) Quantification of LRRK2 and RAB10 pT73 fluorescence intensity on the surface of ingested zymosan particles (LRRK2: *n* = 4; pRAB10: *n* = 5 independent experiments). (**G**) Proteomics volcano plot of WT, G2019S, and N2081D BMDCs treated with zymosan for 30 minutes. (**H**) Venn diagram showing significantly increased proteins after zymosan treatment for each genotype. One-way ANOVA with Tukey’s post hoc test was used for **C** and **F**. *P < 0.05, **P < 0.01, ***P < 0.001, ****P < 0.0001.

DCs are key mediators of DSS-induced colitis that initiate and amplify the inflammatory response (18–20). Several studies have also demonstrated a critical role for LRRK2 in DC biology (7, 19, 21, 22). To investigate the impact of each LRRK2 mutation in primary cells, we isolated BM-derived DCs (BMDCs) from WT, *Lrrk2^G2019S^*, and *Lrrk2^N2081D^* mice. In untreated BMDCs, functional effects of *Lrrk2^G2019S^* and *Lrrk2^N2081D^* mirrored those seen in HEK293 cells, with increased phosphorylation of S1292, RAB12, and RAB10 in *Lrrk2^G2019S^* compared with WT, while the *Lrrk2^N2081D^* variant specifically increased RAB10 phosphorylation (Figure 3A). These results indicate the activation mechanisms identified in HEK293 cells are conserved in primary DCs from LRRK2 KI mouse models.

Given the increased RAB10 phosphorylation observed in *Lrrk2^N2081D^* mutation carriers after severe colonic inflammation, we hypothesized that inflammatory stimuli might activate LRRK2 kinase activity (Supplemental Figure 2, C and D). To test this, BMDCs were stimulated with zymosan, a proinflammatory agent that activates DCs through pattern recognition receptors. IB analysis confirmed activation of the NF-κB pathway across all genotypes. Interestingly, no significant differences were observed in IκBα and IKKβ degradation or in the phosphorylation of NF-κB p65 between genotypes (Supplemental Figure 4). After zymosan treatment, we observed a rapid and striking increase in RAB phosphorylation across all genotypes but with clear LRRK2 mutation-dependent differences in effect size. *Lrrk2^G2019S^* cells exhibited a progressive increase in RAB12 phosphorylation, peaking higher than in WT and N2081D cells. Conversely, N2081D cells had a significant increase in RAB10 phosphorylation relative to G2019S and WT. These findings indicate DC stimulation activates LRRK2 kinase activity, resulting in mutation-dependent differences in substrate phosphorylation (Figure 3, B and C).

To further characterize the activation response, we performed mass spectrometry–based phosphoproteomics and proteomics on DCs. Differentially abundant proteins were calculated relative to untreated WT samples. Phosphoproteome analysis revealed increased phosphorylation of known LRRK2 RAB substrates RAB10 and RAB43, with more modest changes observed in RAB35 and RAB8A after zymosan treatment. The total protein levels of these RAB substrates were consistent across all genotypes. In agreement with our IB data, the relative fold change in RAB10 phosphorylation was higher in zymosan-treated N2081D cells than in WT cells (Figure 3D).

To confirm these findings and explore their mechanistic basis, BMDCs were treated with fluorescently labeled zymosan bioparticles and imaged using confocal microscopy. Zymosan phagocytosis by DCs resulted in the recruitment of LRRK2 to the phagosome surface. Quantification of phosphorylated RAB10 intensity revealed significantly higher accumulation on phagosome surfaces in *Lrrk2^N2081D^* cells compared with WT and *Lrrk2^G2019S^* cells (Supplemental Figure 5 and Figure 3, E and F).

Further analysis of total proteomics data in untreated G2019S and N2081D BMDCs compared with WT revealed few proteins with statistically significant differences in abundance (*q* < 0.05). Similar findings were observed in both brain and colon tissues (Supplemental Figure 6). Upon zymosan stimulation, BMDCs from all genotypes exhibited significant increases in proteins associated with inflammatory signaling relative to the untreated WT baseline. Notably, several of these proteins were more highly elevated in zymosan-treated N2081D cells compared with zymosan-treated WT cells (*P* < 0.05) (Figure 3, G and H). Specifically, we observed increased levels of CCL22, a chemokine involved in recruiting regulatory T cells, and CCR7, a receptor crucial for DC and T cell migration. Additionally, levels of STAT4, a transcription factor essential for Th1 cell differentiation, and TNFSF18, a member of the TNF superfamily involved in T cell costimulation, were elevated. A similar trend was observed in *Lrrk2^G2019S^* cells, though only CCR7 had a statistically significant increase compared with WT.

### LRRK2 kinase inhibitor MLi-2 does not improve colitis in Lrrk2^N2081D^ mice.

We next evaluated potential therapeutic strategies to reduce colitis severity in N2081D mutation carriers, focusing on *Lrrk2^N2081D^* male mice, which exhibited the most severe colitis symptoms among our experimental groups. We hypothesized that kinase inhibitor treatment might mitigate the effects of the N2081D mutation and improve symptoms of DSS-induced colitis. To test this, we provided male *Lrrk2^N2081D^* mutation carriers chow supplemented with MLi-2, a potent and selective LRRK2 inhibitor, targeting a 60 mg/kg/day dose, as previously described by Kluss et al. (23, 24).

*Lrrk2^N2081D^* carriers were provided facility chow or MLi-2–supplemented chow for 3 days prior to DSS treatment. On day 4, we began administering 2% DSS for 8 days, followed by a 3-day recovery period, with mice continuing their respective diets throughout (Supplemental Figure 7, A and B). Colon tissue samples analyzed at the experimental end point showed significant reductions in RAB10 and RAB12 phosphorylation after MLi-2 treatment, confirming effective target engagement (Supplemental Figure 7, C and D). However, MLi-2 had minimal impact on body weight loss, spleen weight, and colon length, damage, and inflammation, suggesting that in-diet dosing of the LRRK2 kinase inhibitor MLi-2 does not provide significant protection from DSS-induced colitis in N2081D mutation carriers (Supplemental Figure 7, E–I).

### Rab12 KO reduces RAB10 phosphorylation and alleviates colitis severity in Lrrk2^N2081D^ mice.

Research from our lab and others has identified RAB12 as a key regulator of LRRK2-dependent RAB10 phosphorylation (25–27). Notably, we recently demonstrated that Rab12 KO prevents LRRK2-G2019S–mediated cilia defects in the mouse brain (27). Whereas *Rab12* KO mice exhibit normal growth and reach adulthood, Rab10 KO mice do not survive past embryonic development (data not shown). We hypothesized that targeting *Rab12* through genetic deletion might mitigate the effects of the N2081D mutation on RAB10 phosphorylation and limit its pathogenic impact. To test this, we crossed *Lrrk2^N2081D^* mice with *Rab12* KO mice. *Lrrk2^N2081D^;Rab12^–/–^* mice appeared grossly normal, with no significant differences in body weight, colon length, or spleen size compared with WT, *Lrrk2^N2081D^*, or *Rab12^–/–^* mice, suggesting loss of *Rab12* is well tolerated (Supplemental Figure 8).

**Figure 4:**
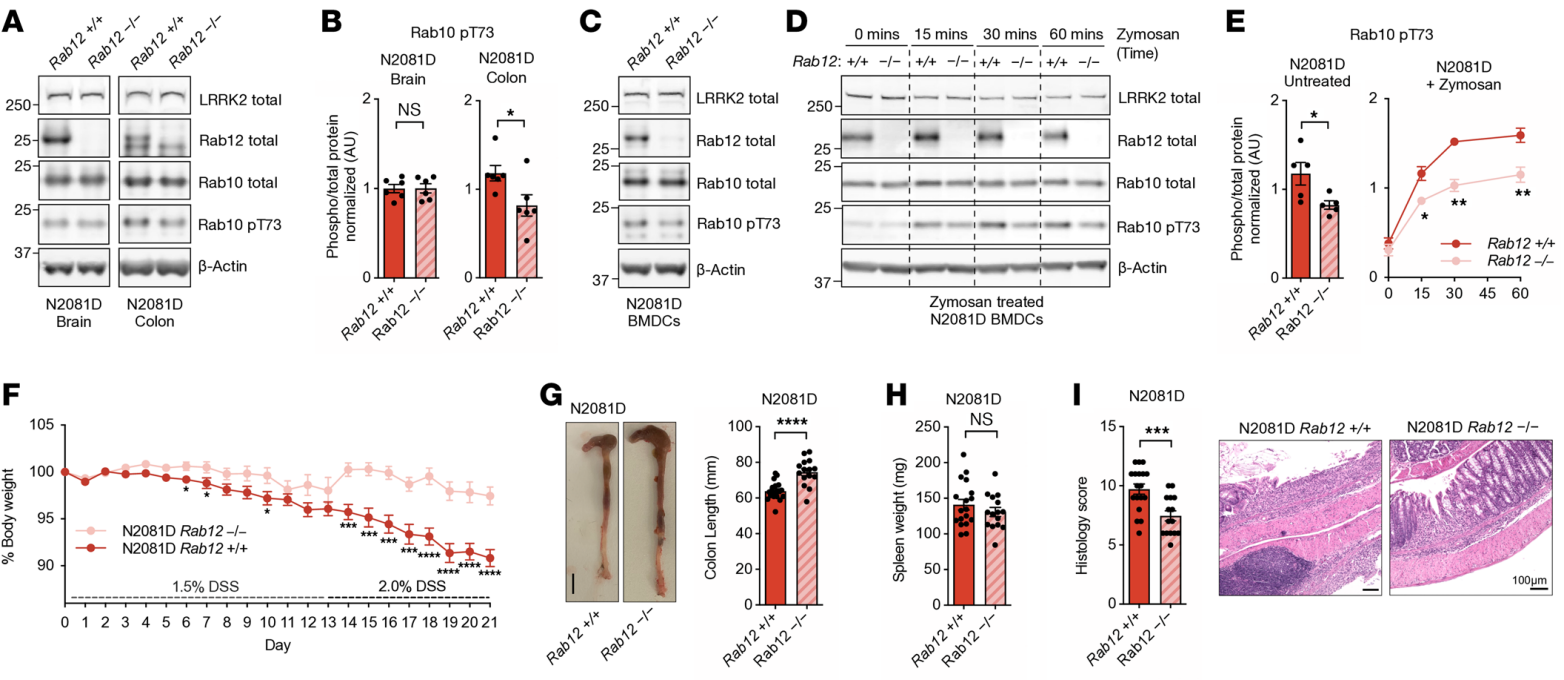
RAB12 KO rescues LRRK2-N2081D phenotypes. (**A** and **B**) Representative IB and quantification of phosphorylated RAB10 in the brain and colon from N2081D and N2081D/RAB12 KO mice. (**C** and **D**) Representative IBs of untreated and zymosan-treated BMDCs from N2081D and N2081D/RAB12 KO mice over 60 minutes (*n* = 4 independent experiments). (**E**) Quantification of IBs from untreated and zymosan-treated BMDCs. (**F**) Normalized body weight of N2081D and N2081D/RAB12 KO animals during 21 days of progressively increasing DSS treatment. (**G**) Representative colon images and colon length measurements at sacrifice. (**H**) Spleen weight at sacrifice. (**I**) Representative histological images and scoring of colon inflammation and damage (*n* = 18 N2081D mice; *n* = 15 N2081D/RAB12 KO mice). Unpaired 2-tailed *t* tests were used for data presented in **B** and **E**–**I**. *P < 0.05, **P < 0.01, ***P < 0.001, ****P < 0.0001.

*Lrrk2^N2081D^;Rab12^–/–^* mice had a significant reduction in phosphorylated RAB10 in colon tissue compared with *Lrrk2^N2081D^;Rab12^+/+^* mice, with only a minor observed effect in the brain (Figure 4, A and B). Reduced RAB10 phosphorylation was also detected in BMDCs of *Lrrk2^N2081D^;Rab12^–/–^* animals compared with *Lrrk2^N2081D^* carriers expressing RAB12 (Figure 4C). Furthermore, *Rab12* KO significantly decreased zymosan-induced N2081D-mediated RAB10 phosphorylation, resulting in a reduced peak signal over 60 minutes (Figure 4, D and E). These findings demonstrate that Rab12 KO effectively reduces LRRK2-N2081D–mediated RAB10 phosphorylation.

We next examined the impact of RAB12 loss on symptoms of induced colitis in *Lrrk2^N2081D^* mice. *Lrrk2^N2081D^;Rab12^–/–^* animals had significantly less body weight loss over the course of DSS administration (Figure 4F). At the experimental end point, *Lrrk2^N2081D^;Rab12^–/–^* animals also had significantly longer colons (Figure 4G). Histological analysis confirmed reduced colonic damage and immune cell infiltration in RAB12 KO mice. A modest reduction in spleen weight was also observed in *Lrrk2^N2081D^;Rab12^–/–^* mice, although this was not statistically significant (Figure 4, H and I).

In summary, we found that deletion of *Rab12* significantly reduces N2081D-associated colitis severity, highlighting its potential as a therapeutic target. Notably, although WT mice experienced less severe DSS symptoms under the same DSS-dosing regimen, they did not show a measurable benefit from *Rab12* KO. This suggests that *Rab12* deletion may have a greater therapeutic benefit specifically in LRRK2-mutation carriers (Supplemental Figure 9).

## Discussion

Our association analysis of CD- and PD-linked LRRK2 variants across multiple biobank data sets underscores the genetic pleiotropy of the LRRK2 gene. Characterizing mice carrying the LRRK2 N2081D risk variant, we found the variant exacerbates induced colitis, confirming its pathogenic role in CD. Consistent with previous reports, we also observed that the LRRK2 G2019S mutation modestly increases colitis severity relative to WT animals (28, 29). We further demonstrate that *Rab12* deletion mitigates the pathogenic effects of LRRK2-N2081D, leading to a significant reduction in intestinal inflammation. These findings establish a LRRK2-linked CD mouse model and identify the RAB12-LRRK2 pathway as a promising therapeutic target for modulating LRRK2 activity in CD treatment.

Our association analysis of *LRRK2* variants with CD and PD was based on publicly available summary statistics, which precluded us from determining whether LRRK2 N2081D is primarily associated with CD in humans in the context of a higher overall burden of common IBD risk alleles. Previous reports have shown strong linkage disequilibrium (LD) between N2081D and PD-associated common variants (30, 31). Although we cannot rule out the possibility that common variants in LD with N2081D contribute to or even drive the risk for both PD and CD, our findings clearly demonstrate involvement of this variant in murine intestinal inflammation and colitis phenotype. Therefore, studies are needed to determine whether the observed association between N2081D and CD is independent of a general genetic predisposition to IBD or reflects a broader polygenic contribution.

Our study also reveals that, unlike LRRK2-G2019S, the N2081D variant specifically enhances the phosphorylation of RAB10 but not RAB12, whereas the G2019S mutation increases phosphorylation of RAB12 and LRRK2-S1292, with a more modest effect on RAB10 in mouse models. These functional differences likely contribute to the diverse phenotypes observed in LRRK2 mutation carriers, leading to varying degrees of IBD symptoms and PD-associated neurodegeneration.

Structurally, the G2019S substitution replaces glycine with serine within the DYG motif of the activation loop in the active site of the kinase domain, increasing its kinetics without significantly altering the overall conformation (17, 18). In contrast, our results suggest the N2081D mutation activates LRRK2 by disrupting an interdomain interaction between the LRR and kinase domains. This conformational shift is crucial for increasing RAB10 phosphorylation, likely by increasing exposure of the kinase active site, but appears to have little effect on the phosphorylation of RAB12. The reasons for this selective effect are not fully understood; however, recent structural analyses suggest RAB12 and RAB10 bind to distinct sites within the armadillo domain of LRRK2 (25, 27). It is possible that the N2081D mutation induces a conformation that preferentially enhances phosphorylation of RABs bound at specific sites on LRRK2 (i.e., RAB10), although this hypothesis requires experimental validation.

In LRRK2-overexpressing HEK293 cells and BMDCs, both N2081D and G2019S variants similarly increased RAB10 phosphorylation. However, zymosan stimulation led to a greater increase in phosphorylated RAB10 in LRRK2-N2081D DCs compared with WT and LRRK2-G2019S cells. Elevated RAB10 phosphorylation was also observed in colon tissues of N2081D mutation carriers versus WT and G2019S KI animals, with further increases seen in carriers experiencing severe DSS symptoms. We are currently unsure which cell types contribute to this effect. Although our study primarily focused on DCs, research examining diverse cell populations under various inflammatory conditions is warranted to determine the mechanisms by which the LRRK2 N2081D mutation enhances colitis severity. Although our data show that the N2081D mutation leads to increased Rab10 phosphorylation, we currently lack direct mechanistic evidence demonstrating that Rab10 itself mediates the enhanced inflammatory phenotype in vivo.

Proteomic analysis suggests elevated RAB10 phosphorylation in LRRK2-N2081D cells coincides with increased inflammatory signaling. This is evidenced by higher levels of proteins associated with immune cell recruitment and activation compared with WT cells after zymosan treatment. Although further confirmation is needed, we propose that these cellular phenotypes likely contribute to the increased severity of colitis observed in LRRK2-N2081D mutation carriers by enhancing immune cell infiltration and sustaining inflammatory responses in intestinal tissue. The precise role of increased RAB10 phosphorylation in this process remains to be determined.

A surprising finding in our study is that dietary administration of the LRRK2 kinase inhibitor MLi-2 did not significantly alleviate DSS-induced colitis in N2081D mice despite reducing RAB10 phosphorylation in colon tissue. Notably, a partial rescue of DSS-colitis severity has been observed in *Lrrk2^G2019S^* mice, using a similar strategy of dietary MLi-2 dosing (32). Additionally, LRRK2 inhibitors have been shown to reduce zymosan-evoked increases in TNF-α and other proinflammatory cytokines in the supernatant of mouse and human cell cultures (19). These findings suggest that alternative experimental approaches, such as testing mice of different ages or sexes, varying the LRRK2 genotype, adjusting the DSS dosing regimen, using germ-free versus pathogen-free conditions, or modifying kinase inhibitor strategies (e.g., using different structural inhibitors, changing doses, altering the route of administration) might yield a stronger therapeutic effect. It is also possible that the N2081D mutation confers increased sensitivity to colitis through a kinase-independent mechanism, rendering kinase inhibitors insufficient as a therapeutic strategy. One potential way to address this possibility is to generate an N2081D KI mouse model bearing a secondary mutation that inactivates the kinase domain. Finally, there is concern that MLi-2 may have off-target effects that could interfere with its ability to rescue colitis. Consequently, we advise caution when interpreting these data. Nonetheless, our findings suggest kinase inhibition alone may not be sufficient to counteract the heightened sensitivity to induced colitis caused by the N2081D mutation.

In contrast, our study showed that Rab12 deletion significantly reduced phosphorylated RAB10 levels in colon tissue and led to a marked improvement in colitis severity across multiple readouts. However, it is important to note that the MLi-2 and Rab12 KO DSS experiments were conducted in separate cohorts and at different times. Additionally, the Rab12 KO mice were younger (12 weeks old vs. 16 weeks old) and received a more gradual DSS regimen—starting at 1.5% and escalating to 2.0%—over a longer duration (21 days vs. 8 days plus a 3-day recovery). These experimental differences may contribute to the observed variation in therapeutic effects.

That said, we propose that Rab12 may play an important role in mediating inflammatory pathways in *Lrrk2^N2081D^* mice and could represent a promising target for therapeutic development. We also found that *Rab12* KO lowered levels of phosphorylated RAB10 in colon tissue and BMDCs, both under basal conditions and following zymosan-induced activation. Although the N2081D variant does not increase RAB12 phosphorylation beyond WT levels, our data indicate N2081D-induced RAB10 hyperphosphorylation depends on the presence of RAB12 and likely its interaction with LRRK2 (27). While the mechanisms by which RAB12 KO regulates LRRK2 function are still being resolved, recent studies in cellular models have shown that RAB12 depletion disrupts LRRK2-induced perinuclear clustering of lysosomes and impairs LRRK2’s recruitment to damaged lysosomal membranes (26). Our recent work also demonstrated that the deletion of RAB12 can reverse the reduced cilia formation and increased incidence of split centrosomes caused by LRRK2-G2019S in astrocytes (27).

Based on our findings, we propose that RAB12 depletion could significantly mitigate the pathogenic effects of the LRRK2 N2081D mutation in vivo. However, the mechanism by which RAB12 loss influences LRRK2-mediated inflammatory pathways remains poorly understood. Our results highlight this as a critical area for further investigation.

## Methods

### Sex as a biological variable.

This study included both male and female mice in our colitis modeling experiments, where we observed sexually dimorphic effects. For tissue and BMDC analyses, only male mice were used, to ensure consistency in experimental conditions.

### Association analysis of LRRK2 variants with CD and PD in 3 large biobanks.

Bio*Me* Biobank genotyping was performed using the Infinium Global Screening array (Illumina) and Infinium Global Diversity (Illumina) arrays. The Global Screening array and Global Diversity data were processed, quality checked, and imputed together using the TOPMeD-r2 reference panel (33). To determine genetic ancestries of Bio*Me* participants, the genotypes were combined with the 1000 Genomes Project phase 3 (34) reference panel (https://www.cog-genomics.org/plink/2.0/resources; primary release, build 38, *n* = 3,202 samples). To select the optimal number of ancestral populations (K), ADMIXTURE (35) was employed to calculate cross-validation errors for K values from 4 to 12, and GrafPOP (36) was used to assign samples to a superpopulation. The Frobenius distance was used to compare each ADMIXTURE matrix *K* with the GrafPOP matrix, determining *K* = 10 as the best fit for aligning self-identified and genetically determined matrices. The genetically determined classes have been unified to ancestry groups by identifying 1000 Genomes Project reference samples with the highest ancestral proportions in each class.

Principal component analysis was performed using LD-pruned variants (*r*^2^ = 0.2) with a minor allele frequency greater than 5% and not exceeding Hardy-Weinberg equilibrium with a *P* < 1 × 10^−6^ using Plink, version 2 (37). Patients with CD or PD were identified using electronic health records containing *International Classification of Diseases, 10th Revision* (*ICD-10*) and *ICD-9* diagnoses of Bio*Me* participants matched with genotype data. Patients with CD were identified using K50 (*ICD-10*) and 555 (*ICD-9*) codes; patients with PD were identified using G20 (*ICD-10*) and 332 (*ICD-9*) codes.

Genebass (https://app.genebass.org/) provides exome-based association statistics of 394,841 UK BioBank participants (12). “We downloaded single-variant association statistics from the UK Biobank Google Cloud bucket (gs://ukbb-exome-public/500k/results/variant_results.mt) by following the instructions at https://app.genebass.org/downloads.) Associations of the G2019, N2081D, and N551K variants with CD (*ICD-10* code K50) and PD (*ICD-10* code G20) were extracted using Hail (https://github.com/hail-is/hail/commit/678e1f5).

Associations of the G2019, N2081D, and N551K variants with CD and PD were extracted from ancestry-specific and meta-analysis results available through the MVP PheWeb browser (https://phenomics.va.ornl.gov/web/cipher/pheweb) (13).

### Association testing and meta-analysis of LRRK2 variants.

Population-specific association testing was conducted in the Bio*Me* BioBank African American (AFR), admixed American (AMR), and European American (EUR) cohorts using the scalable and accurate implementation of generalized mixed model (SAIGE) (38). Analyses were adjusted for age, biological sex, array type, and the first 10 principal components (PCs 1–10) as covariates. For meta-analysis, we used METAL software (39) to perform both European-specific and multi-ancestry meta-analyses, using the inverse-variance weighted method.

### Plasmids.

Christian Johannes Gloeckner (University of Tübingen, Tübingen, Germany) provided the human LRRK2 pDEST-NSF-tandem affinity plasmid. We generated additional LRRK2 variants using the QuikChange Lightning Site-Directed Mutagenesis Kit, following the manufacturer’s instructions (Agilent, 210518).

### HEK cell culture, transfection, and lysis.

HEK293 cells were cultured in DMEM (Gibco) containing 10% FCS, 100 U/mL penicillin, and 100 μg/mL streptomycin at 37°C in a humidified incubator with 5% CO_2_. Cells were seeded in 6-well plates and transiently transfected at ~75% confluency with 2 μg of DNA using Lipofectamine 2000, per the manufacturer’s instructions (ThermoFisher Scientific). After 24 hours, cells were lysed in ice-cold 1× Cell Lysis Buffer (CLB; Cell Signaling, 8903) supplemented with 1× protease and phosphatase inhibitors (ThermoFisher, 78440) and left on ice for 30 minutes. Cell lysates were clarified by centrifugation at 15,000*g* for 10 minutes at 4°C, and protein concentration was determined using the Pierce BCA Protein Assay Kit (ThermoFisher, 23228). We loaded 15 μg of protein for quantitative IB analysis.

### Structural analysis and graphic display.

The structural model of LRRK2 (Protein Data Bank identifier 7LI4) was manually examined using the *Coot* package (40) and PyMol (41) to glean the potential interactions occurring at N551 and N2081. The potential dynamics of these residues were analyzed using Nanoscale Molecular Dynamics (NAMD) software (42). The Chemistry at Harvard Macromolecular Mechanics (CHARMM) force field was used to parameterize the simulation. The system was solvated inside a box of water molecules with a 13 Å padding in each direction. The system was then neutralized with 0.15 M NaCl. Simulation was performed with a 1 fs time step. The minimized structure was heated from 0 to 300 K over 300 picoseconds. The production run was performed in the NVE (microcanonical) ensemble at 300 K. The total simulation time was 2 ns, and coordinates were recorded every 1 picosecond. Structural graphic presentations were made with PyMol.

### Generation of LRRK2 N2081D KI mouse model.

The N2081D missense mutation was incorporated into the murine LRRK2 gene using gene editing (CRISPR/Cas9) technology as described previously (43). In brief, 1 gRNA (Integrated DNA Technologies [IDT]) binding to the genomic target (GCTAACTCATCAAACTCATTGG) was selected after the evaluation of its off-target potential using the web-based tool CRISPOR (http://crispor.tefor.net) and its on-target efficiency by electroporating gRNA/Cas9 reagents (2 μM) into 0.5-day fertilized zygotes, followed by sequence analysis of target genomic region of 2-cell stage embryos. The synthetic single-stranded editing template (IDT) contains the N2081D missense mutation, and the flanking homology arm is as follows: 5′CTTACTACTTCACGATATTTGGACAACTGGGAGTAGGATTATGGAGGGTTTGAGGTTCCCAGATGAATTCGATGAGTTAGCCATACAAGGGAAGTTGCCAGGTAAGTTCTGGTTTTATCTACAAGAGTTCTTTTCTTAATGTCAGCTTGGTCATGTAGAG.

Two silent mutations were also incorporated into the editing template to create a diagnostic EcoRI restriction enzyme recognition site and to prevent the recut by the gRNA/Cas9 DNA nuclease. The single-stranded donor DNA (10 ng/μL) was co-microinjected with preassembled gRNA/Cas9 riboproteins (0.2 μM gRNA, 0.2 μM Cas9 protein; IDT) into the pronuclei of mouse zygotes on a C57BL/6N background. The resulting founder animals were subsequently backcrossed with C57BL/6J mice.

### Animals.

The G2019S KI mouse line was provided by Dianna Benson (Icahn School of Medicine at Mount Sinai, New York, New York, USA). RAB12 KO mice were generated by our group in a previous study (27) by deleting exon 3 of the *Rab12* gene in the C57BL/6J background using CRISPR/Cas9 technology. To generate N2081D with RAB12 KO mice, N2081D homozygotes were crossed with RAB12 KO mice. These mice were then bred to produce RAB12 KO and N2081D homozygotes. In experiments requiring genotype-, age-, and sex-matched cohorts, parallel breeding pairs for each genotype (N2081D, G2019S, and WT) were established. Litters were born within days of each other, enabling the formation of age- and sex-matched groups that were cohoused under a 12-hour light/dark cycle in the same animal colony.

### Tissue lysis.

Organ tissue samples were collected, immediately frozen on dry ice, and stored at –80°C until lysis. Frozen tissues were homogenized in ice-cold 1× homogenization buffer (20 mM Tris-HCl, 0.5 mM EDTA, 250 mM sucrose) supplemented with 1× protease and phosphatase inhibitors (ThermoFisher, 78440). Tissues were placed in screw-cap RINO microcentrifuge tubes containing stainless steel beads (NextAdvance) and homogenized using the Bullet Blender Gold (NextAdvance). The homogenate was combined with 10× CLB to achieve a 1× final concentration and rotated at 4°C for 30 minutes. Tissue lysates were centrifuged at 15,000*g* for 10 minutes at 4°C, and supernatants were quantified using the Pierce BCA Protein Assay Kit (ThermoFisher, 23228). For IB analysis, 50 μg of protein per sample was loaded.

### DSS administration to induce colitis.

To induce colitis, 2% (w/v) DSS (molecular weight 36,000–50,000 Da; MP Biomedicals) was dissolved in drinking water and provided ad libitum. The DSS solution was freshly prepared and replaced every 2–3 days for consistent dosing. Mice were 16 weeks old when DSS effects on each type (WT, G2019S, and N2081D) were compared and 12 weeks old when N2081D mice and RAB12 KO effects were compared. Mice whose body weight loss exceeded 20% of their original weight were euthanized as a humane end point.

Mice were monitored daily for signs of colitis, such as weight loss, stool consistency, and rectal bleeding. At the end of the DSS treatment or upon reaching humane end points, mice were euthanized, and colonic tissues were collected for histological and molecular analyses to assess colitis severity and inflammatory responses. Histological scoring was performed using a modified scale (excluding regeneration assessment) as described by Lamas et al (44). Stool samples were analyzed for Lipocalin-2 levels using ELISA (R&D Systems, DY1857).

### IB analysis.

Samples were mixed with 4× SDS-PAGE loading buffer supplemented with 150 mM DTT and heated at 95°C for 5 minutes. They were loaded onto NuPAGE 4%–12% Bis–Tris Midi Gels (Thermo Fisher Scientific, WG1402BOX or WG1403BOX) and electrophoresed at 120 V in NuPAGE MOPS SDS running buffer (Thermo Fisher Scientific, NP0001-02). After electrophoresis, proteins were transferred onto a nitrocellulose membrane (GE Healthcare, Amersham Protran-supported 0.45 mm nitrocellulose) at 20 V for 60 minutes in transfer buffer (25 mM Tris base, 190 mM glycine, 15% methanol). The membranes were blocked with Intercept TBS blocking buffer (LicorBio) at room temperature for 1 hour. They were incubated overnight at 4°C with the primary Ab in TBS blocking buffer mixed with TBS-T (50 mM Tris base, 150 mM NaCl, 0.1% Tween 20). Membranes were washed in TBS-T before incubation with the secondary Ab (IRDye, LicorBio) according to the manufacturer’s instructions. After secondary Ab incubation, membranes were washed in TBS-T, and protein bands were detected using the Odyssey Classic Imaging System and quantified using Image Studio Lite.

### Culturing of BMDCs.

Mouse femurs and tibias were flushed with a 23G needle using ice-cold PBS. Cells were pelleted by gentle centrifugation and resuspended in Red Blood Cell Lysis Buffer (eBioscience, 00-4300-44) for 3 minutes, then diluted with PBS and strained through a 40 μm filter (Greiner EASYstrainer, 542040). After pelleting, cells were resuspended in RPMI 1640 medium containing 10% FCS, 100 U/mL penicillin, and 100 μg/mL streptomycin, supplemented with 20 ng/mL GM-CSF (PeproTech) and 20 ng/mL IL-4 (PeproTech). On day 3, half of the medium was replaced. On day 6, cells were replated in supplemented RPMI medium and used for experiments on day 7. Cells were stimulated with RPMI medium containing Zymosan (InvivoGen) at 0.1 mg/mL for the indicated times. For confocal imaging, cells were treated with Zymosan A BioParticles, Alexa Fluor 594 conjugate (ThermoFisher, 723374), and processed as described later in *Confocal microscopy*.

### Phosphoproteomics and global proteomics sample preparation.

Proteomics samples were first lysed with 100 μL of lysis buffer [60 mM tetraethylammonium bromide, pH 8.5, 5 mM Tris(2-carboxyethyl)phosphin, and 25 mM 2-chloroacetamide in 10% acetonitrile (ACN)] for 30 minutes at 76°C with agitation (1,200 rpm) on an Eppendorf Thermomixer C. Afterward, proteins were digested with trypsin and LysC (Sigma Aldrich) with a 1:100 protein/enzyme ratio overnight at 37°C and with agitation (1,200 rpm). Formic acid (FA) was added to a final concentration of 1% to stop the digestion. Samples were placed in a SpeedVac at 30°C and spun under vacuum until full dryness. Samples were reconstituted in 0.1% FA.

For global proteomics, 200 ng of peptides were subjected to Evotips purification (described in the next paragraph). For phosphopeptide enrichment, 200 μg of peptides in 100 μL were subjected to enrichment using the default phosphopeptide-enrichment protocol from the AssayMAP Bravo Platform (Agilent). For the enrichment, 50 mL of 0.1% FA in ACN was used as the priming buffer, 50 mL of 0.1% FA in 80% ACN as the equilibration buffer, and 50 mL of 500 mM ammonium hydrogen phosphate as the elution buffer with Fe(III)-NTA cartridges (Evosep) (for additional information, see ref. 45) Evotips were first soaked in 1-propanol for 3 minutes, then washed 2 times with 0.1% FA in 100% ACN (EvoB), soaked for 3 minutes in 1-propanol, and then washed again 2 times with 0.1% FA in water (EvoA), all at 700*g* for 1 minute. EvoA (70 μL) was loaded on Evotips at 700*g* for 15 seconds before samples were loaded at 700*g* for 1 minute. Evotips were washed with 50 μL of EvoA. An additional 150 μL of EvoA was loaded on top at 700*g* for 15 seconds. Samples were then subjected to liquid chromatography–mass spectrometry (LC-MS) analysis.

### Data-independent acquisition LC-MS analysis.

For the global proteomics experiment, samples were subjected to LC–tandem MS (LC-MS/MS) analysis on a Orbitrap Astral mass spectrometer (Thermo Fisher Scientific) coupled online to an Evosep One LC system. Peptides were eluted from the Evotips with up to 35% ACN and separated using a 21-minute gradient for a throughput of 60 samples/day on an Aurora Rapid TS column of 8 cm, 150 μm internal diameter with 1.7 μm C18 beads (IonOpticks). Column temperature was maintained at 50°C using a column oven (IonOpticks). The Orbitrap Astral MS was equipped with a FAIMS Pro interface and runs were acquired using a FAIMS compensation voltage of –40 V and a total carrier gas flow of 3.5 L/min. Full MS scans was in the range of 380–980 mass/charge ratio with an Orbitrap resolution of 120,000 with a normalized automated gain control of 500% and a maximum injection time of 3 ms. For Astral MS/MS scans in data-independent mode, the isolation windows were set to 3 Th with a maximum injection time of 5 ms and an automated gain control target of 800%. Isolated ions were fragmented using HCD with a normalized collision energy of 25%.

For phosphoproteomics analysis, we used a timsTOF Pro 2 mass spectrometer (Bruker) coupled to the Evosep One system. Peptides were eluted from Evotips with up to 35% ACN and separated using a 44-minute gradient for 30 samples/day on a PepSep C18 column (15 cm × 150 μm, 1.5 μm; Bruker). The dia-PASEF method consisted of 8 dia-PASEF scans, which incorporated 2 ion mobility windows per dia-PASEF scan (cycle time 2.7 seconds). The method covered a mass-to-charge range of 400 to 1,400 and an ion mobility range from 0.75 to 1.45 Vs/cm^2^. For all experiments, we used accumulation and ramp times of 100 ms. We implemented a constantly decreasing collision energy profile from 60 eV at 1.5 Vs/cm^2^ to 54 eV at 1.17 Vs/cm^2^ to 25 eV at 0.85 Vs/cm^2^ and ending at 20 eV at 0.6 Vs/cm^2^.

### Bioinformatics analysis.

For the global proteomics experiment, data-independent acquisition (DIA) raw files were processed using the library free search in DIA-NN 1.8.1 (46) against a UniProt mouse reference proteome of canonical sequences with 17,214 entries, where the enzyme specificity was set to trypsin, with a maximum of 1 missed cleavage site, a maximum peptide length of 30, and minimum of 7, up to 1 variable modification, and carbamidomethylation as a fixed modification, and the oxidation of methionine was set as a variable modification. The deep learning–based spectra mode, FASTA digest for library free search, and retention times (RTs) and ion mobility (IM) prediction with heuristic protein inference were enabled. FDR control for the precursor was set to 1% and the remaining settings were set to default.

For the phosphopeptide experiment, DIA raw files were processed using directDIA in Spectronaut against the same Uniprot mouse reference proteome. Default settings were used with the following exceptions: variable modification was set to Phospho (STY) with the 3–25 best fragments per peptide, normalization filter type set to phospho (STY), and a probability cutoff at 0. The peptide table was retrieved in a suitable format for the peptide collapse plugin described in Perseus (47). Peptide collapse was conducted with the default setting except for the aggregation type, which was set to sum.

For the bioinformatics analysis, Python, version 3.5.5, with the *pandas* 1.4.2, *numpy* 1.21.5, *matplotlib* 3.5.13, *seaborn* 0.11.2, *scipy* 1.7.3, and *statsmodels* 0.13.2 packages were used. Protein and peptide intensities were first normalized using directLFQ and then log_2_-transformed. Data were filtered for valid values in at least 1 experimental group and imputed using a sampling method with a shifted Gaussian normal distribution (width = 0.3 and downshift = 1.8). Statistical significance was determined using an unpaired 2-sided Student’s *t* test. *P*-value correction was done using the Benjamini-Hochberg method.

### Antibodies.

The Abs used for IB analysis were as follows: mouse mAb against FLAG peptide (Sigma, F1804; 1:5,000), mouse mAb against β-actin (Cell Signaling, 4967; 1:5,000), rabbit mAb against LRRK2 (Abcam, MJFF2, ab133474, 1:2,000), rabbit mAb against LRRK2 phospho-S1292 (Abcam, ab203181; 1:1,000), rabbit mAb against total-RAB10 (Abcam, ab237703; 1:2,000), and rabbit mAb against RAB10 phospho-T73 (Abcam, ab230261; 1:1,000). For immunocytochemistry, the Abs used were rabbit mAb against RAB10 phospho-T73 (Abcam, ab230261; 1:100) and rabbit mAb against LRRK2 (Abcam, MJFF2, ab133474; 1:100).

### Confocal microscopy.

Cells were seeded on coverslips and washed with PBS-T. They were fixed in 4% paraformaldehyde in PBS for 15 minutes at room temperature. The cells were blocked with 1% BSA (Sigma-Aldrich) in PBS plus 0.1% Triton X-100 for 30 minutes. Primary Abs were diluted in 1% BSA in PBS plus 0.1% Triton X-100 and incubated at 4°C overnight. After washing 3 times with PBS-T, the cells were incubated with secondary Abs for 60 minutes at room temperature in the dark. The coverslips were mounted on glass slides with ProLong Gold Antifade Mountant with DAPI (Life Technologies). Slides were imaged using an LSM 900 microscope.

### Statistics.

Statistical differences in HEK293 cell experiments were calculated using 1-way ANOVA with Dunnett’s multiple comparison test, comparing each group with the WT. Comparisons among WT, G2019S, and N2081D samples were conducted using 1-way ANOVA with Tukey’s post hoc test for multiple comparisons. For comparisons between 2 experimental groups, an unpaired 2-tailed *t* test was used.

### Study approval.

All procedures were conducted in accordance with guidelines approved by the IACUC at the Icahn School of Medicine at Mount Sinai.

### Data availability.

All data supporting the findings of this study are included in the Supporting Data Values file. Additional materials are available from the corresponding author upon reasonable request, under standard institutional material transfer agreements.

## Author contributions

GRH, IP, and ZY conceptualized the study, devised the methodology, and acquired funding for the work. GRH, Xiangting Li, XZ, YZ, DTV, MO, OK, QQH, MEK, MW, LT, and MM all contributed to the investigation. GRH provided visualization of the data and wrote the original draft of the manuscript. ZY supervised the study. GRH, YZ, DTC, OK, QQH, MEK, and IP reviewed and edited the manuscript. GRH, Xingjian Li, and NK contributed revisions to the manuscript.

## Supplementary Material

Supplemental data

Unedited blot and gel images

Supplemental tables 1-2

Supporting data values

## Version 1 10/01/2025

Electronic publication
