# Supplementary material for: Targeting specific kinase substrates rescues increased colitis severity induced by the Crohn’s disease–linked LRRK2-N2081D variant
Source: J Clin Invest. 2025 Oct 1;135(19):e190017. doi: 10.1172/JCI190017 (PMC12483564; doi:10.1172/JCI190017)
Supplement: Supplemental data [file jci-135-190017-s005.pdf]

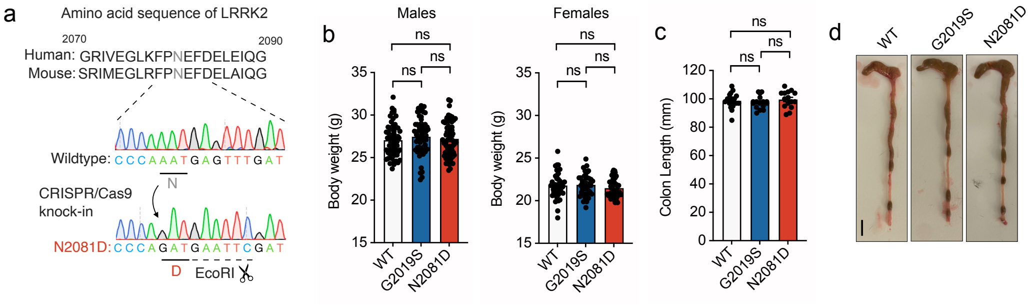

Supp. Fig. 1 | Generation and phenotypic evaluation of LRRK2-N2081D mice. (a) Human and mouse amino acid sequence alignment of the LRRK2 kinase domain, with nucleotide sequencing of wild-type and N2081D knock-in animals. A silent EcoRI diagnostic site was introduced to identify the N2081D allele. (b) Body weight of 16-week-old male and female mice (n males: WT 60, G2019S 58, N2081D 62; n females: WT 44, G2019S 42, N2081D 44). (c) Colon length measurements from WT, G2019S knock-in, and N2081D knock-in 16-week-old male mice (n animals: WT 19, G2019S 16, N2081D 15). (d) Representative photograph of colons. One-way ANOVA with Tukey's post hoc test was used for panels b and c. *p < 0.05, **p < 0.01, ***p < 0.001, ****p < 0.0001.

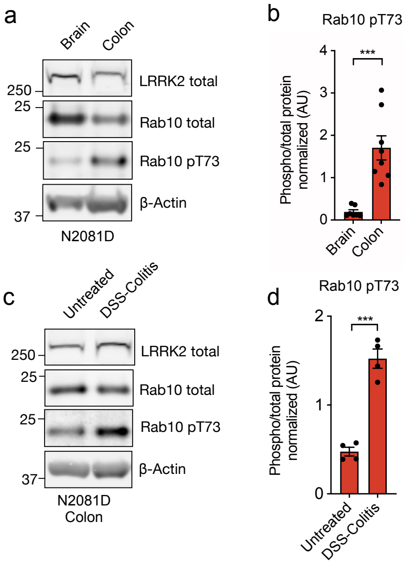

Supp. Fig. 2 | Tissue analysis of RAB10 phosphorylation in LRRK2-N2081D mutation carriers. (a, b) Representative immunoblot and quantification of phosphorylated RAB10 in brain and colon tissue from LRRK2-N2081D animals. (c, d) Representative immunoblot and quantification of phosphorylated RAB10 in colon samples from untreated LRRK2-N2081D mice and LRRK2-N2081D mice with severe colitis symptoms. Unpaired two-tailed t-tests were used for panels b and d. *$p < 0.05$, **$p < 0.01$, ***$p < 0.001$, ****$p < 0.0001$.

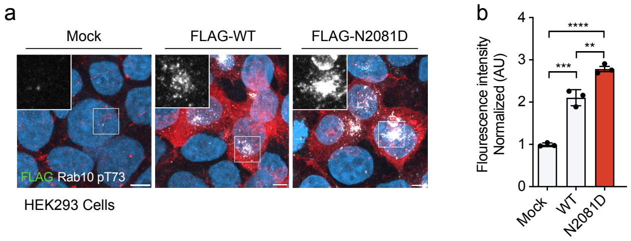

Supp. Fig. 3 | Imaging quantification of transfected HEK293 cells. (a) Representative confocal images showing LRRK2 transfection and phosphorylated RAB10. (b) Quantification of phosphorylated RAB10 fluorescence intensity (n = 3 independent experiments). One-way ANOVA with Tukey's post hoc test was used for panel b. *p < 0.05, **p < 0.01, ***p < 0.001, ****p < 0.0001.

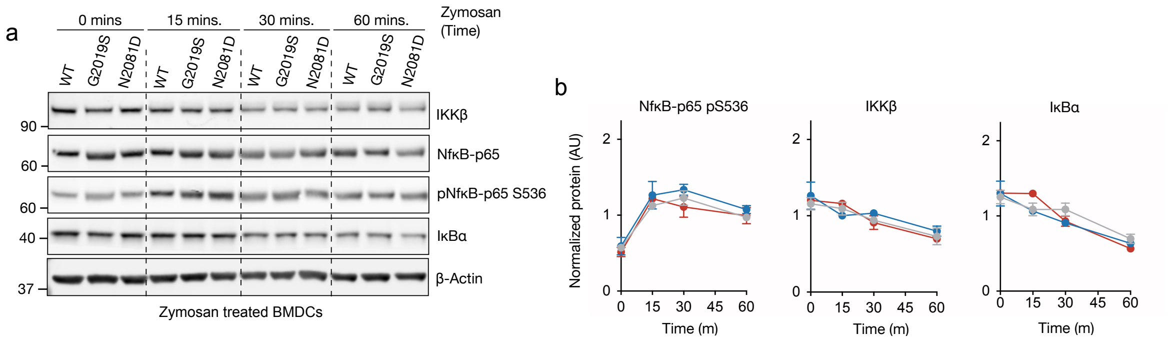

Supp. Fig. 4 | Zymosan treatment activates the NF-κB pathway. (a) Representative immunoblot of WT, G2019S, and N2081D BMDCs treated with zymosan over 60 minutes (n = 4 independent experiments). (b) Quantification of immunoblots from zymosan-treated BMDCs. One-way ANOVA with Tukey's post hoc test was used for panel b.

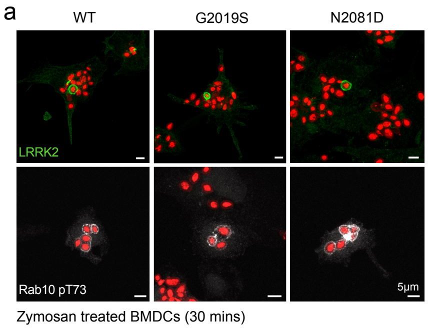

Supp. Fig. 5 | Confocal images of BMDCs treated with zymosan. (a) Representative confocal images of BMDCs used to quantify endogenous LRRK2 and phosphorylated RAB10 levels following 30 minutes of zymosan treatment.

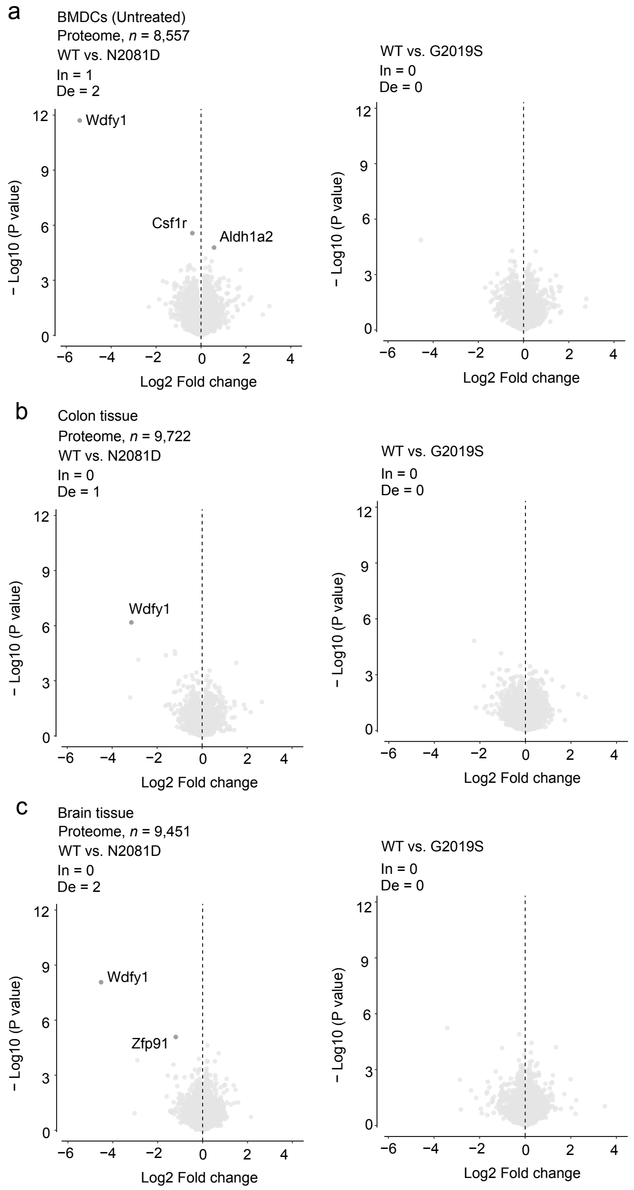

Supp. Fig. 6 | Proteomics Volcano Plots of WT, G2019S, and N2081D Samples. (a) Volcano plot comparing untreated BMDCs from G2019S and N2081D mice to WT. **(b)** Volcano plot comparing colon tissue from G2019S and N2081D mice to WT. **(c)** Volcano plot comparing brain tissue from G2019S and N2081D mice to WT (animals *n*, WT:8 G2019S:8 N2081D:8 used for BMDC cultures, 8 individual experiments).

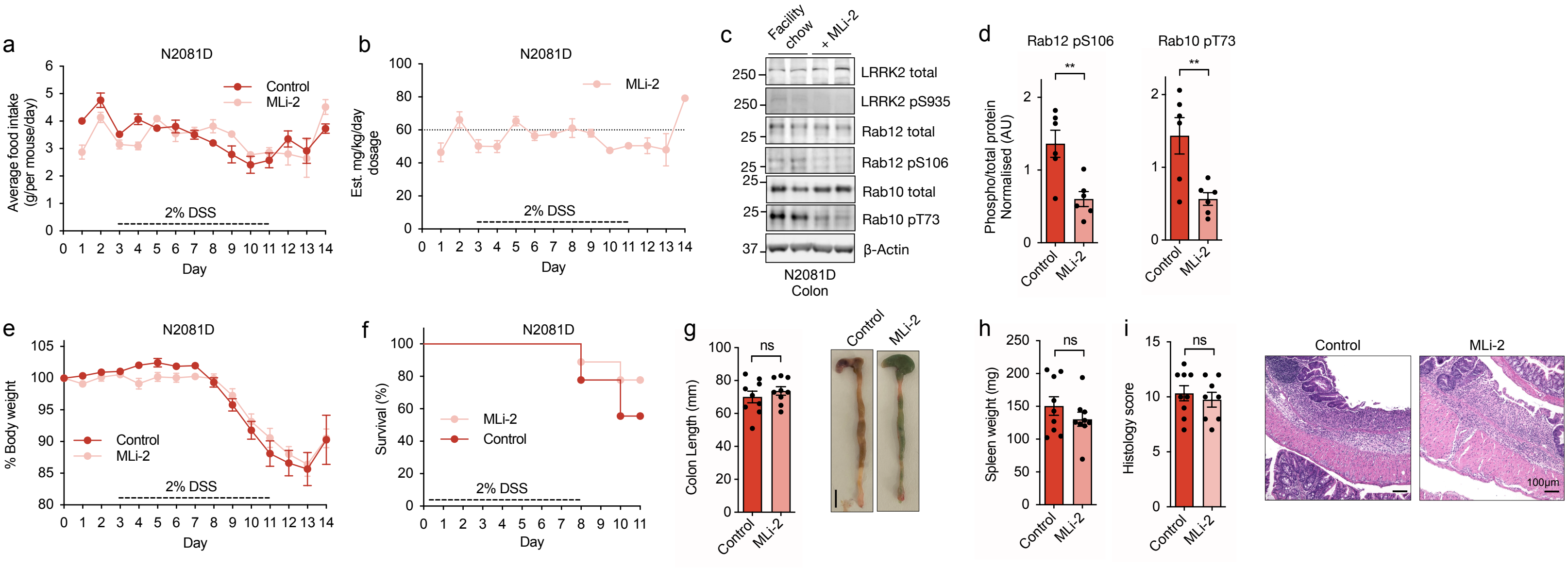

Supp. Fig. 7 | Treatment of LRRK2-N2081D animals with LRRK2 kinase inhibitor MLi-2 supplemented chow. (a) Average food intake per cage of N2081D mice on facility chow or MLi-2 supplemented chow. (b) Estimated MLi-2 dosage in mg/kg/day. (c, d) Representative immunoblot of colon tissue from N2081D mice and quantification of LRRK2 Rab substrate phosphorylation. (e) Normalized percentage of body weight during DSS treatment. (f) Survival curves for LRRK2-N2081D males; animals surpassing humane endpoints were sacrificed. (g) Representative colon images and colon length measurements at sacrifice. (h) Spleen weight recorded at sacrifice. (i) Representative histological images and scoring (n animals: Control 9, MLi-2 9). Unpaired two-tailed t-tests were used for panels a, b, d, e, h, and i. Log-rank (Mantel–Cox) test was used for panel f. $*p < 0.05$, $**p < 0.01$, $***p < 0.001$, $****p < 0.0001$.

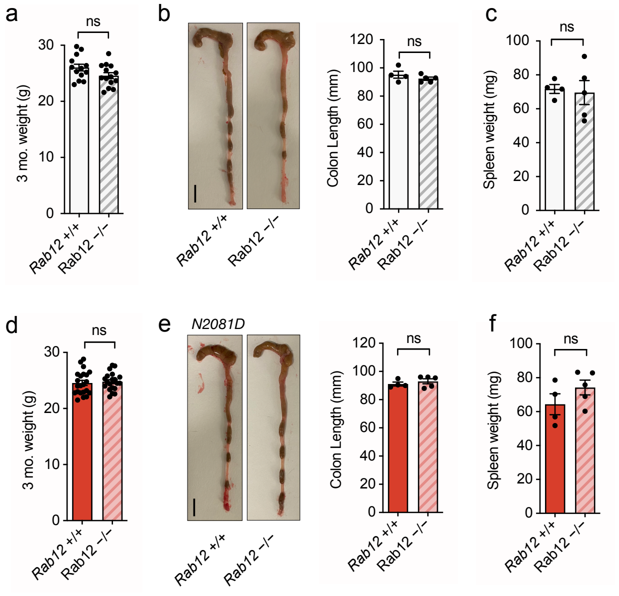

Supp. Fig. 8 | Assessment of RAB12 knockout on body weight, colon length, and spleen weight. (a) Body weight at 3 months of age in WT and RAB12 KO male mice (n animals: WT 13, RAB12 KO 14). (b) Representative colon images and colon length measurements in WT and RAB12 KO male mice. (c) Spleen weight in WT and RAB12 KO male mice (n animals: WT 4, RAB12 KO 5). (d) Body weight at 3 months of age in N2081D and N2081D/RAB12 KO male mice (n animals: N2081D 22, N2081D/RAB12 KO 20). (e) Representative colon images and colon length measurements in N2081D and N2081D/RAB12 KO male mice. (f) Spleen weight in N2081D and N2081D/RAB12 KO male mice (n animals: N2081D 4, N2081D/RAB12 KO 5). Unpaired two-tailed t-tests were used for all panels. *p < 0.05, **p < 0.01, ***p < 0.001, ****p < 0.0001.

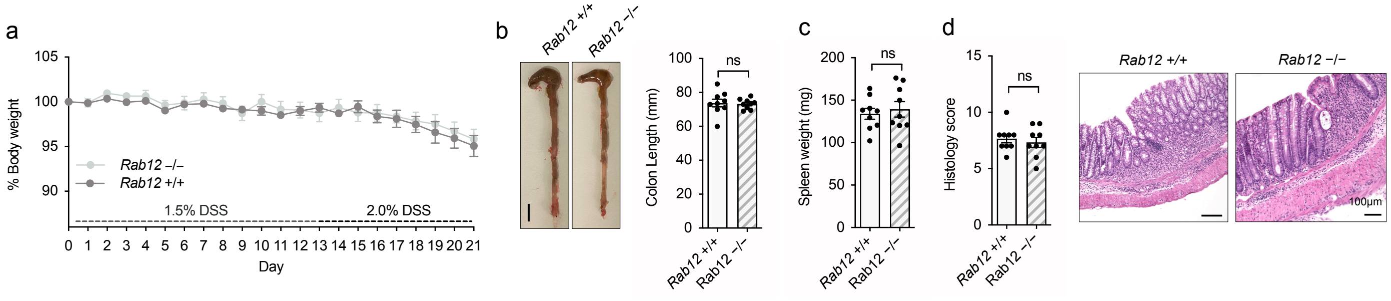

Supp. Fig. 9 | Assessment of WT and RAB12 KO animals' response to DSS-induced colitis. (a) Normalized body weight of WT and RAB12 KO animals during progressively increasing DSS treatment over 21 days. (b) Representative colon images and colon length measurements at sacrifice. (c) Spleen weight of WT and RAB12 KO animals at sacrifice. (d) Representative histology images and scoring (n animals: WT 9, RAB12 KO 9). Unpaired two-tailed t-tests were used for all panels. *p < 0.05, **p < 0.01, ***p < 0.001, ****p < 0.0001.

.
