# Supplementary material for: Targeting specific kinase substrates rescues increased colitis severity induced by the Crohn’s disease–linked LRRK2-N2081D variant
Source: J Clin Invest. 2025 Oct 1;135(19):e190017. doi: 10.1172/JCI190017 (PMC12483564; doi:10.1172/JCI190017)

The red boxes on each blot or gel indicate the region that was cropped and used in the corresponding figure panel in the manuscript. No other modifications were applied.

## Figure 2

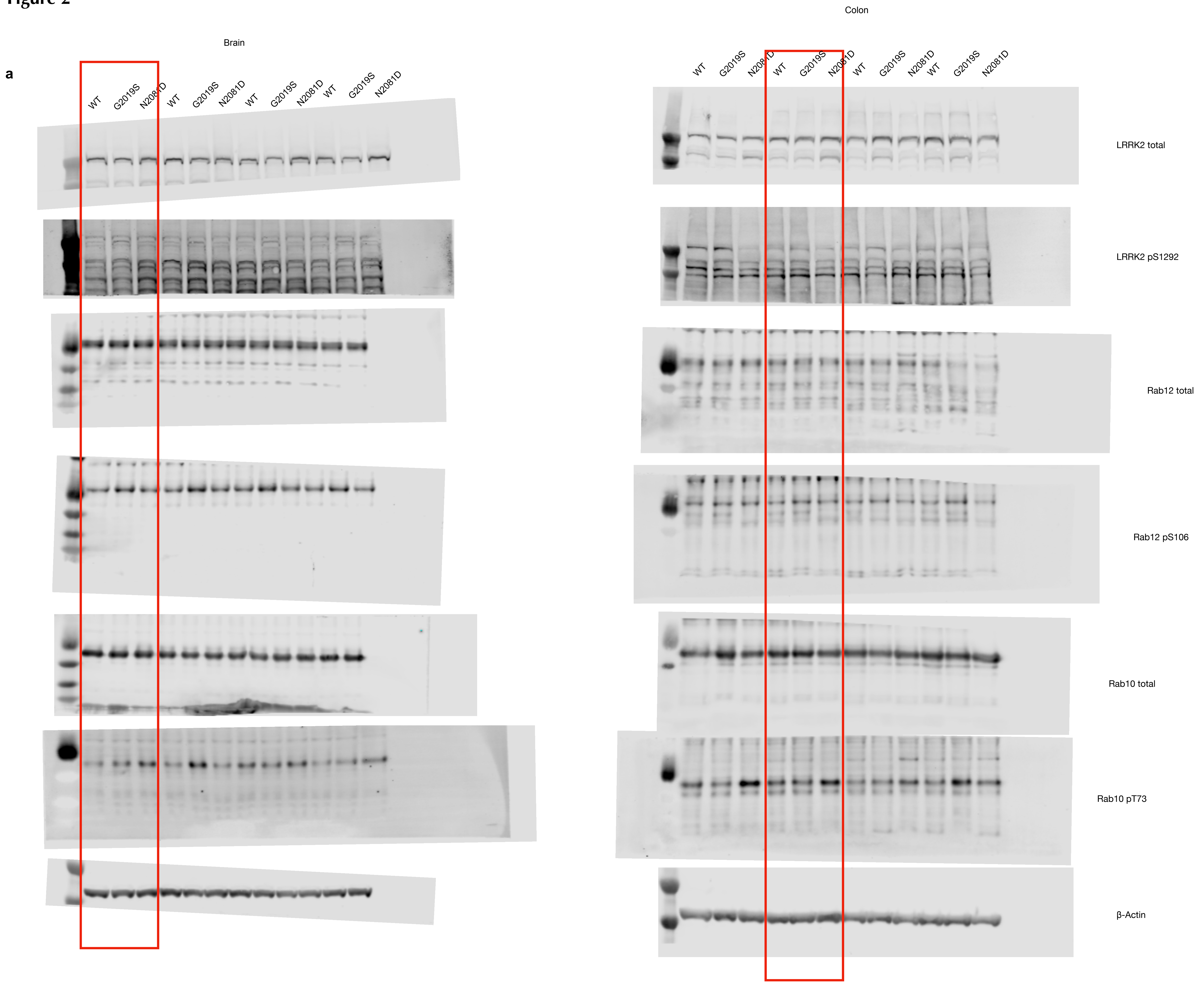

## Figure 2

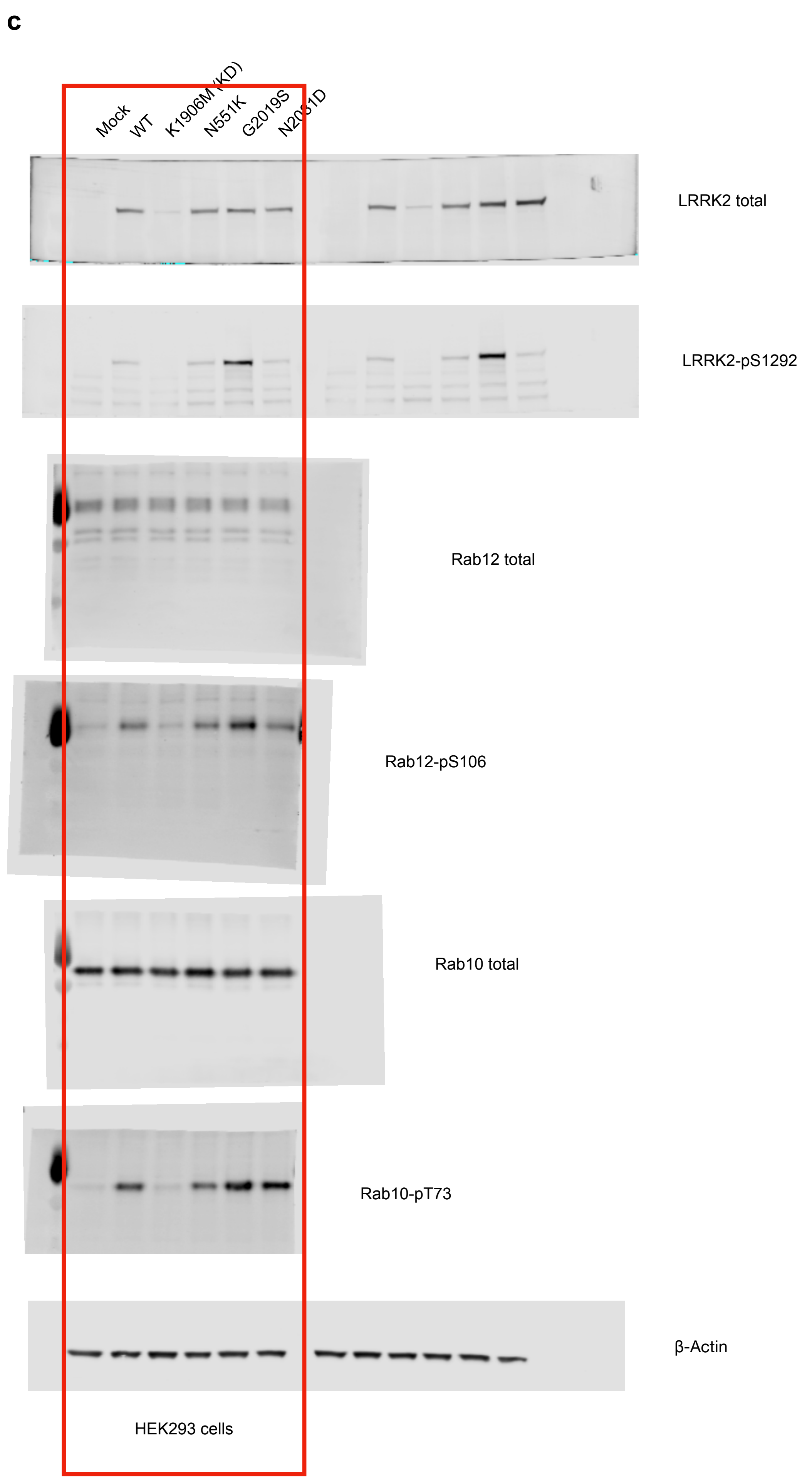
c
Mock
WT
K1906M (KD)
N551K
G2019S
N2081D
LRRK2 total
LRRK2-pS1292
Rab12 total
Rab12-pS106
Rab10 total
Rab10-pT73
β-Actin
HEK293 cells

Figure 2

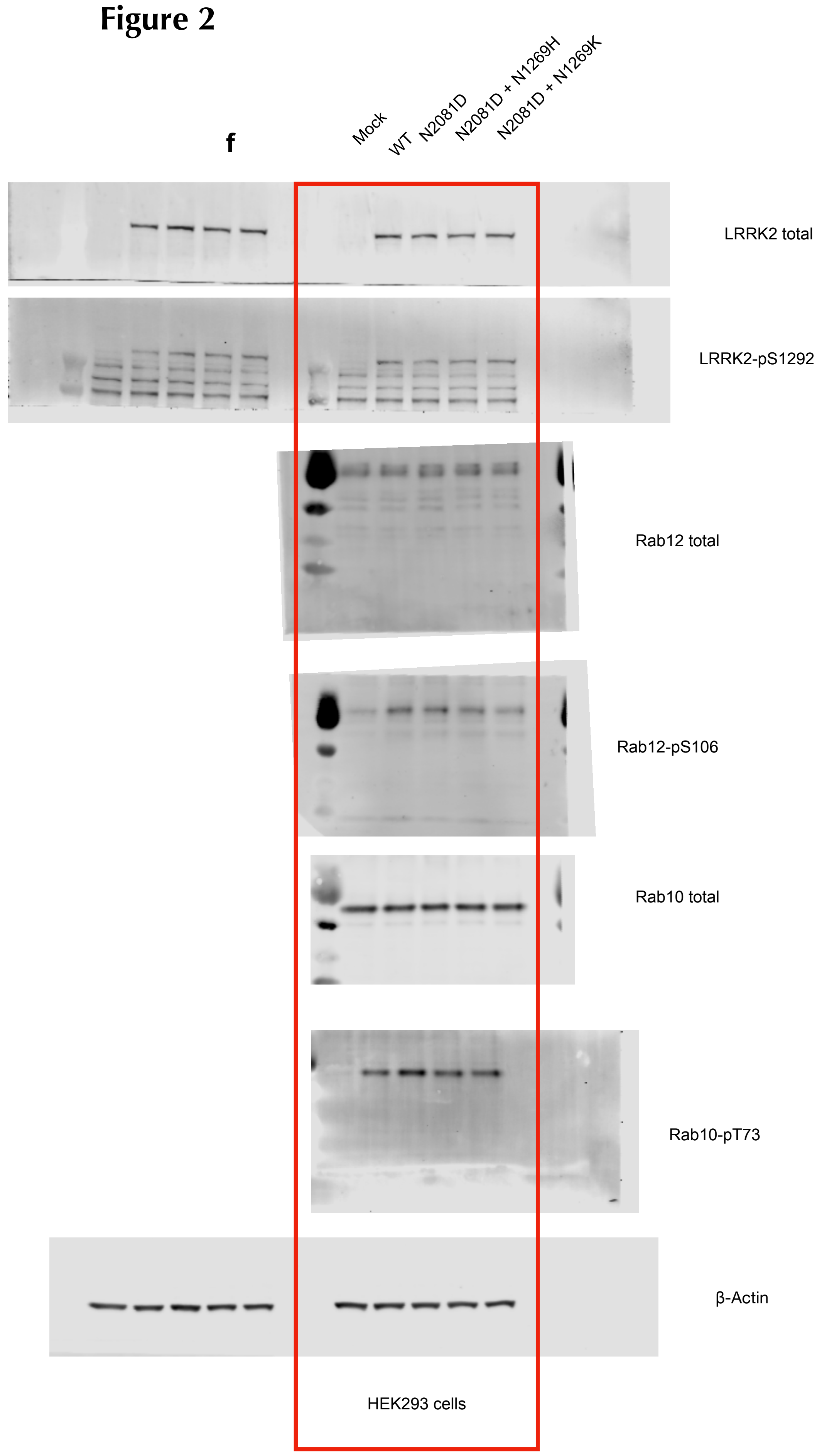

f
Mock
WT
N2081D
N2081D + N1269H
N2081D + N1269K
LRRK2 total
LRRK2-pS1292
Rab12 total
Rab12-pS106
Rab10 total
Rab10-pT73
β-Actin
HEK293 cells

**Figure 3**

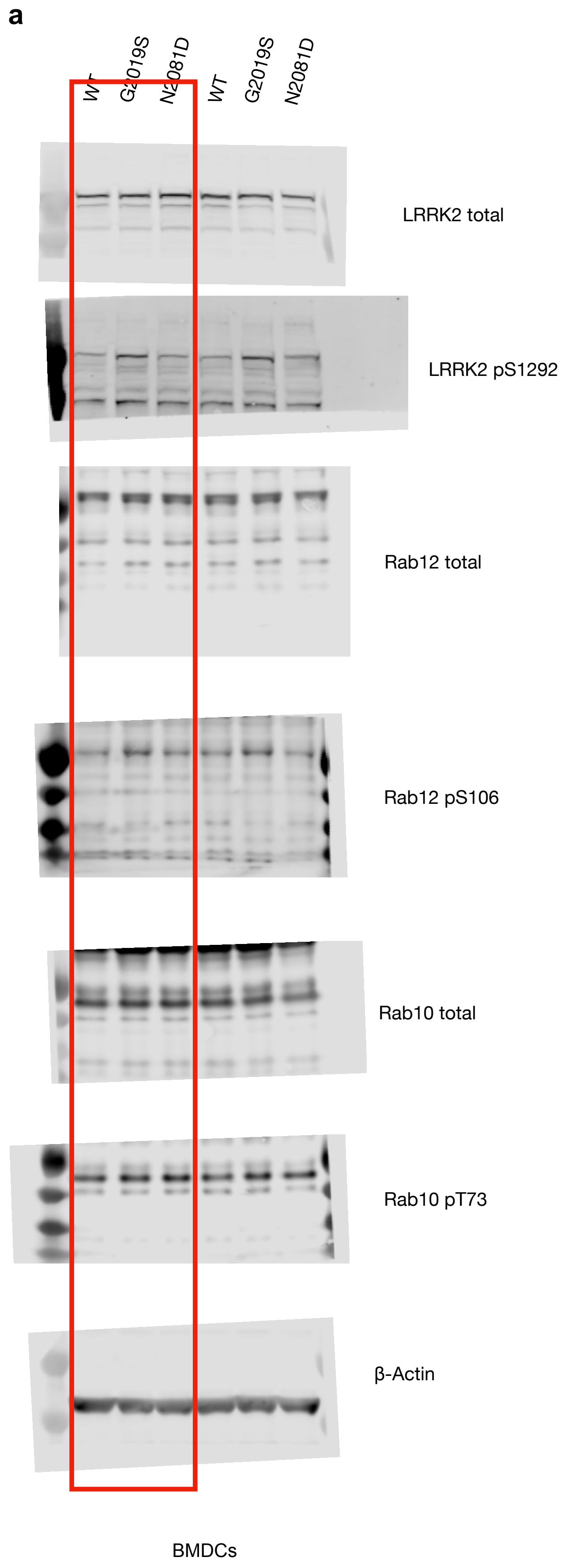
a
WT
G2019S
N2081D
WT
G2019S
N2081D
LRRK2 total
LRRK2 pS1292
Rab12 total
Rab12 pS106
Rab10 total
Rab10 pT73
β-Actin
BMDCs

**Figure 3**

**b**

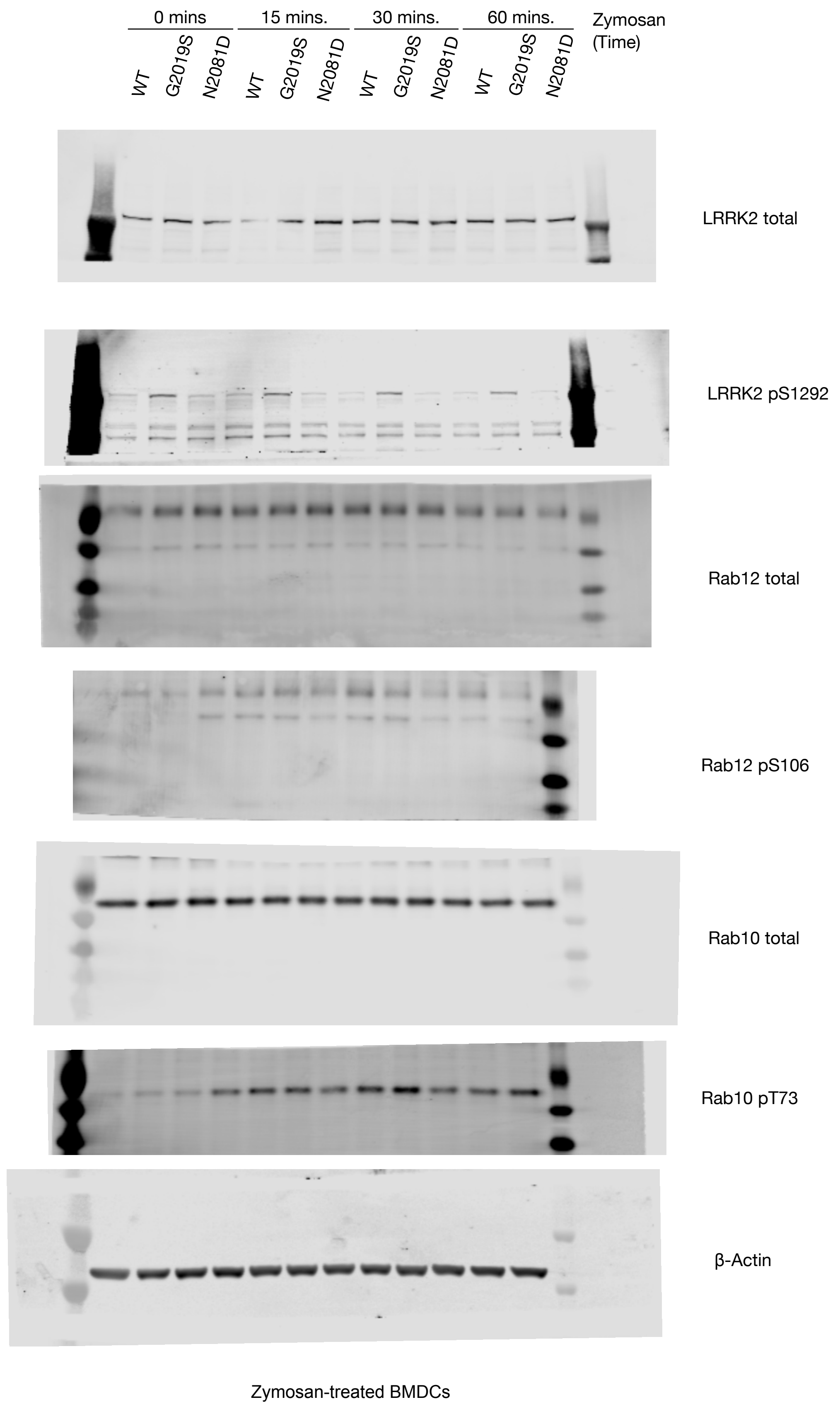
0 mins
15 mins.
30 mins.
60 mins.
Zymosan (Time)
WT
G2019S
N2081D
LRRK2 total
LRRK2 pS1292
Rab12 total
Rab12 pS106
Rab10 total
Rab10 pT73
β-Actin
Zymosan-treated BMDCs

**Figure 4**

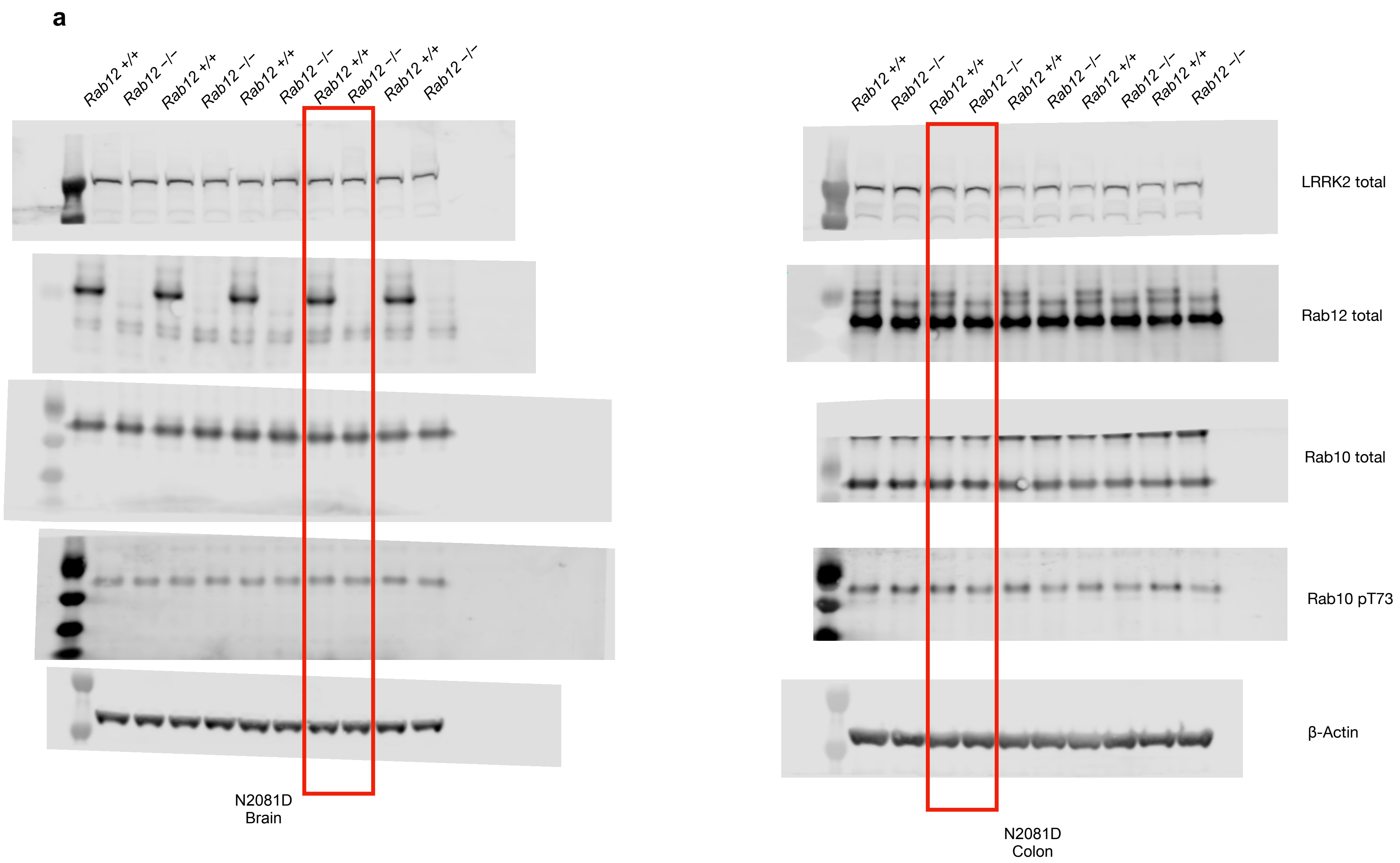
a
Rab12 +/+
Rab12 –/–
Rab12 +/+
Rab12 –/–
Rab12 +/+
Rab12 –/–
Rab12 +/+
Rab12 –/–
Rab12 +/+
Rab12 –/–
N2081D
Brain
Rab12 +/+
Rab12 –/–
Rab12 +/+
Rab12 –/–
Rab12 +/+
Rab12 –/–
Rab12 +/+
Rab12 –/–
Rab12 +/+
Rab12 –/–
LRRK2 total
Rab12 total
Rab10 total
Rab10 pT73
β-Actin
N2081D
Colon

## Figure 4

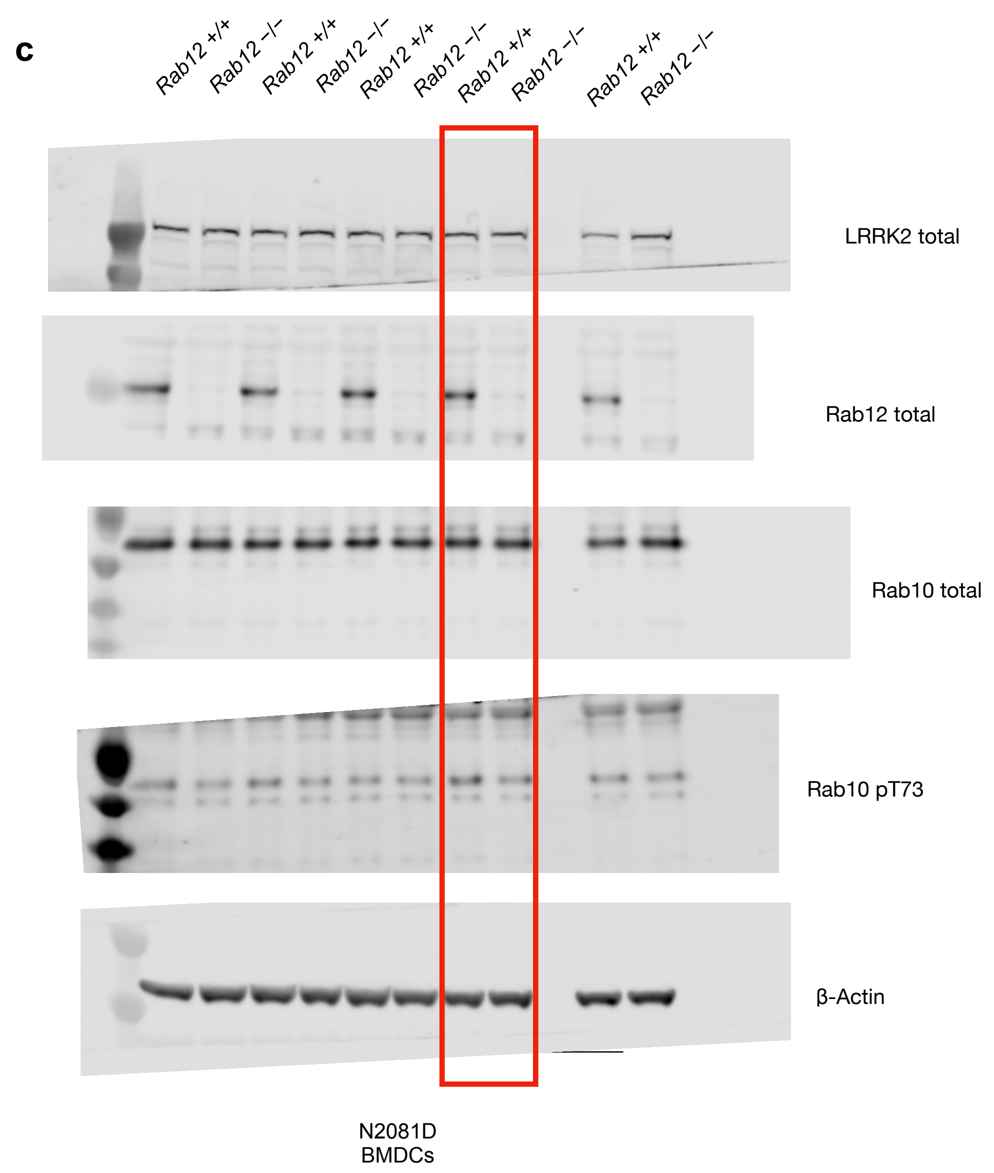
c
Rab12 +/+
Rab12 –/–
Rab12 +/+
Rab12 –/–
Rab12 +/+
Rab12 –/–
Rab12 +/+
Rab12 –/–
Rab12 +/+
Rab12 –/–
LRRK2 total
Rab12 total
Rab10 total
Rab10 pT73
β-Actin
N2081D
BMDCs

# Figure 4

d

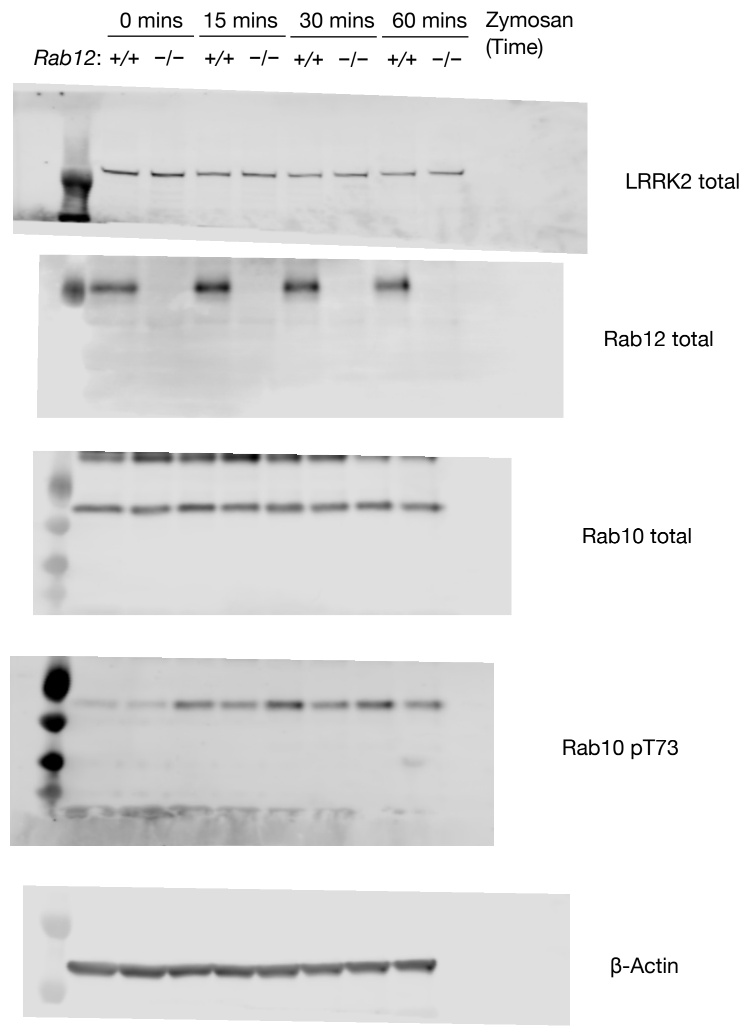


Zymosan treated
N2081D BMDCs

**Supplementary Figure 2**

**a**

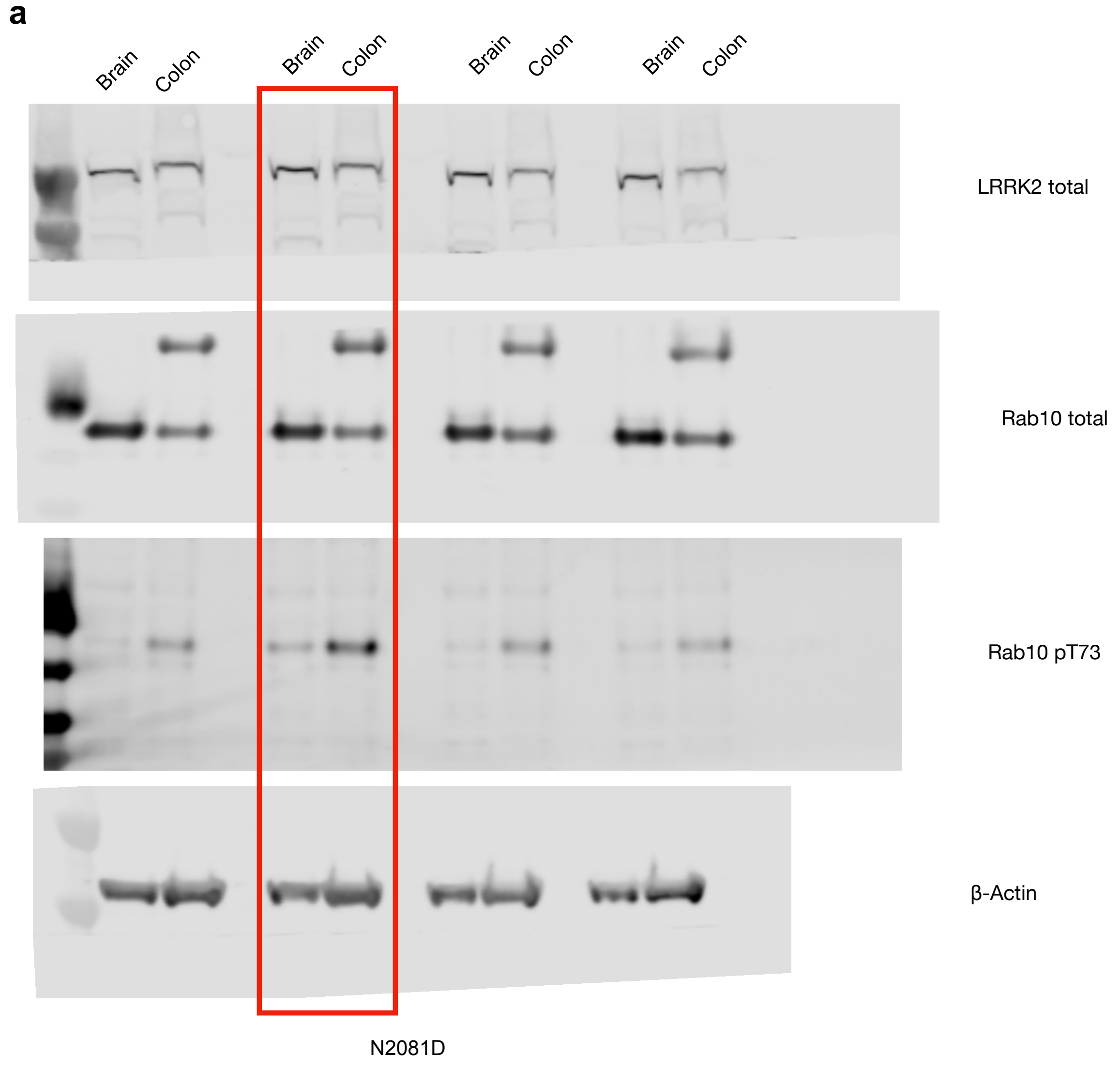

## Supplementary Figure 2

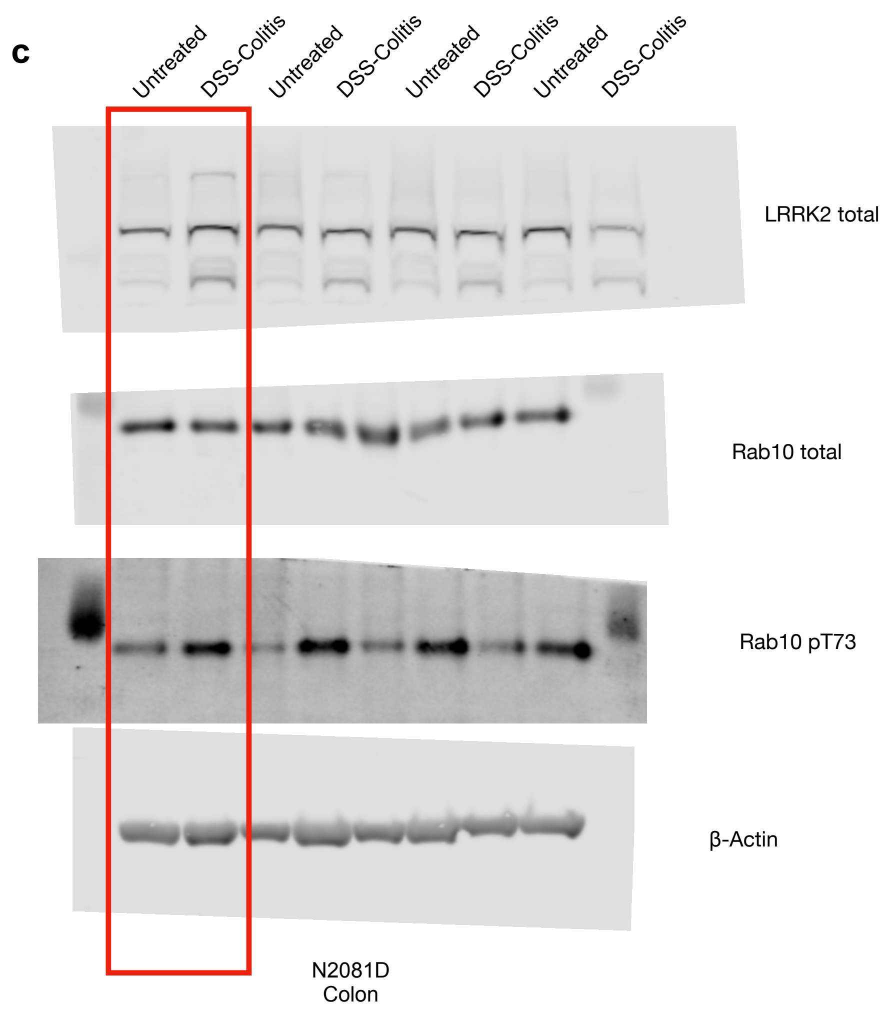

## Supplementary Figure 4

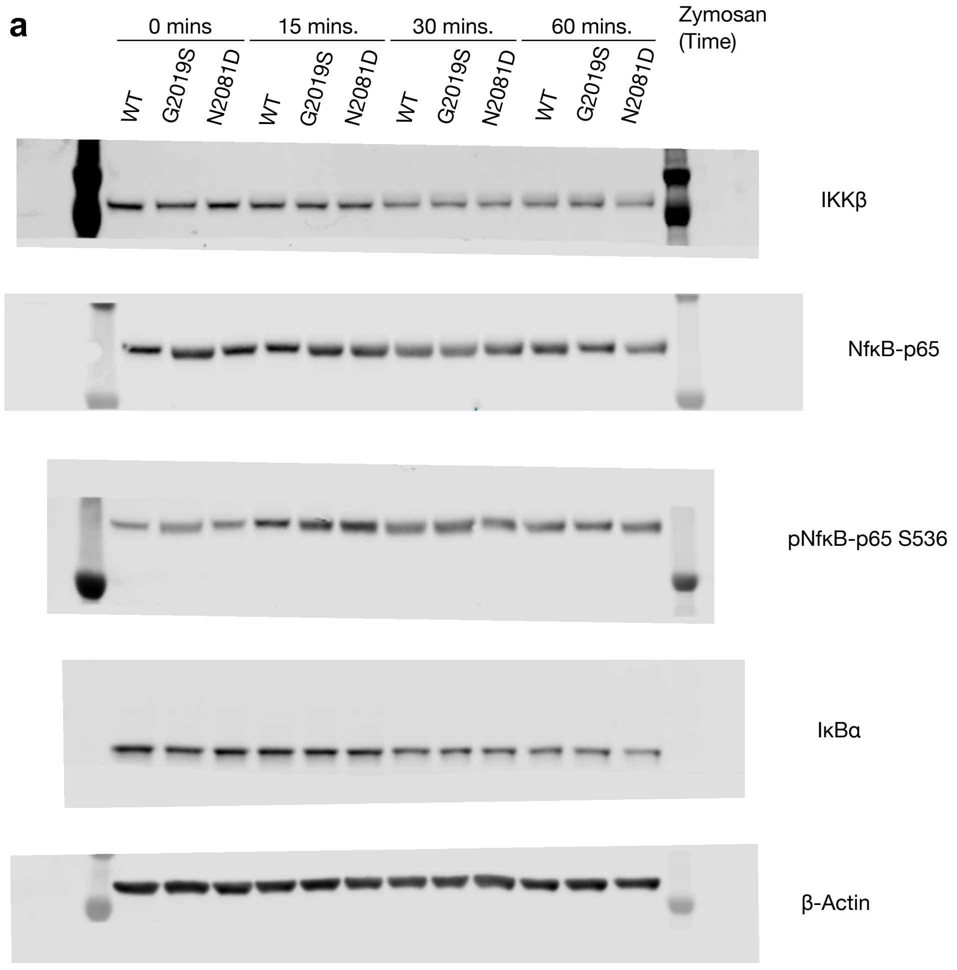


Zymosan treated BMDCs

# Supplementary Figure 7

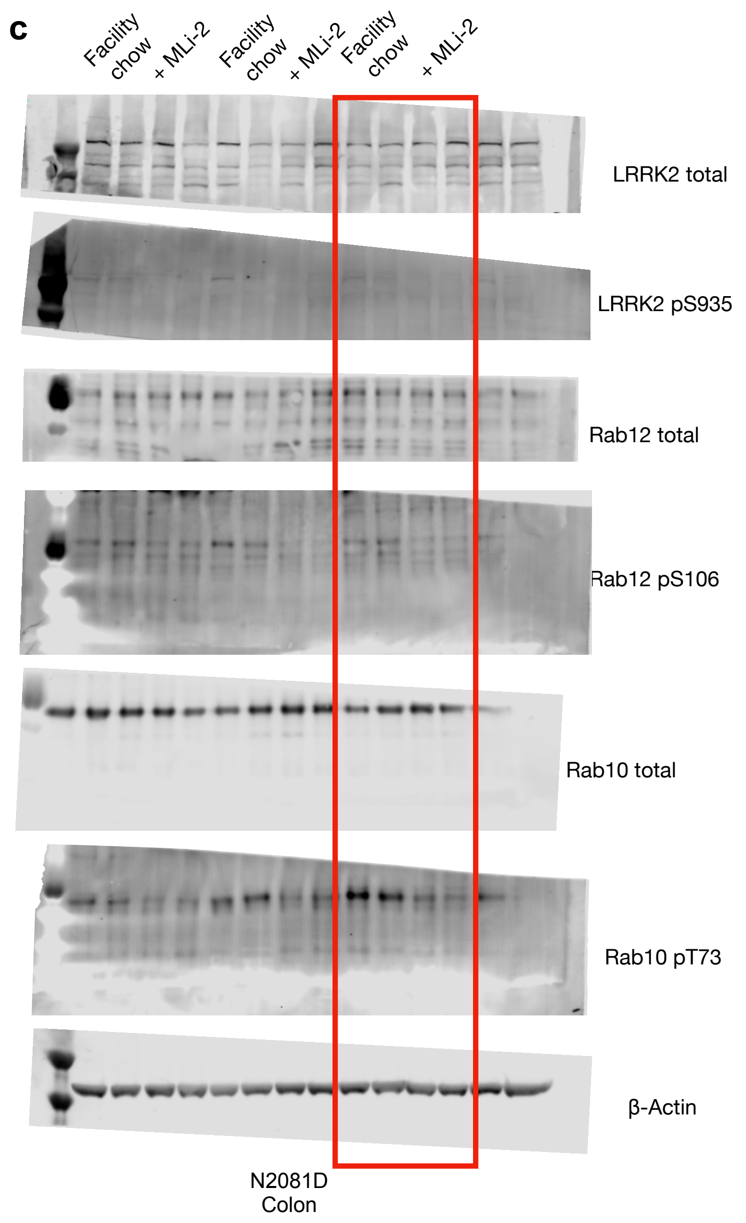

Supplement: Unedited blot and gel images [file jci-135-190017-s006.pdf]
